# Identifying unmet palliative care needs of nursing home residents: A scoping review

**DOI:** 10.1371/journal.pone.0319403

**Published:** 2025-02-25

**Authors:** Patrice Crowley, Mohamad M. Saab, Isabel Ronan, Sabin Tabirca, David Murphy, Nicola Cornally

**Affiliations:** 1 Catherine McAuley School of Nursing and Midwifery, University College Cork, Cork, Ireland; 2 School of Computer Science and Information Technology, University College Cork, Cork, Ireland; Laval University, CANADA

## Abstract

**Introduction:**

Many nursing home residents do not receive timely palliative care despite their need and eligibility for such care. Screening tools as well as other methods and guidelines can facilitate early identification of nursing home residents unmet palliative care needs.

**Aim:**

To map and summarise the evidence on identifying unmet palliative care needs of nursing home residents.

**Methods:**

Any paper reporting on nursing home residents’ unmet palliative care needs were eligible for inclusion. CINAHL, MEDLINE, Embase, Web of Science, APA PsycINFO, and APA PsycArticles and grey literature were systematically searched over two months, February and March 2024. Data were extracted using data extraction forms. Data were synthesised using descriptive analysis and basic content analysis.

**Results:**

Forty six records were included in this review. Nineteen methods, five screening tools, and four guidelines related to identifying residents unmet palliative care needs were identified. Most methods such as the Minimum Data Set and Palliative Care Needs Rounds were implemented as part of an intervention. Limited evidence was identified on what methods healthcare professionals use in daily practice. In total, 117 non-disease specific indicators for identifying residents unmet palliative care needs were identified, with physical indicators such as pain and weight loss being the most represented.

**Conclusion:**

While developments have been made related to the concept of ‘unmet palliative care needs’, a clear definition is required. Evidence-based standardisation of methods for identifying unmet palliative care needs would ensure timely and equitable access to palliative care for nursing home residents worldwide. Achieving this goal requires incorporating screening for unmet palliative care needs into routine care.

## Introduction

Palliative care uses a comprehensive approach to care with priority given to improving an individual’s quality of life. The focus of palliative care is not on curative treatment rather on symptom relief and addressing an individual’s needs [1]. Palliative care is provided to people living with a life limiting condition such as cancer, organ failure, dementia, multiple sclerosis, among others [2]. In 2020, the World Health Organization [3] reported a lack of palliative care provision worldwide; resulting in a deficiency in the provision of palliative care across all healthcare settings.

The four core domains of palliative care needs are: physical, psychological, spiritual, and social needs [1,3]. However, Goni-Fuste et al. [4] recommended the addition of several new domains such as information provision, autonomy, and personal affairs which should also be considered when completing a comprehensive palliative care needs assessment. A primary component transcending all four domains is the early identification of an individual’s needs [1,3]; timely identification facilitates earlier implementation of palliative interventions. Benefits of implementing palliative care early include better management of symptoms, access to external services, reduced unplanned hospital admissions, and improved quality of life [5–7].

The concept of unmet palliative care needs has varying or limited definitions within the literature [8–10]. Ventura et al. [11] suggested this may be due to a presumed common understanding of the words “unmet” and “need”. Considering the suggestion by Ventura at al [11]. as well as the core domains of palliative care needs, we defined an unmet palliative care need as an unfulfilled physical, psychological, social, or spiritual need(s) [1,3,12]. These associated needs must have the potential to be fulfilled through palliative care for example, with effective symptom management, initiation of advance care planning, or referral for hospice care.

Stephens et al. [13] evaluated the provision of palliative care in three American nursing homes. Of the 157 residents deemed eligible for palliative care, none were receiving it. Furthermore, ten Koppel et al. [14] examined palliative care in long-term care in six countries from Europe. They found that when palliative care was implemented, it was usually started in the last two weeks of the resident’s life which limits the benefit of receiving such care.

Nursing home residents are a unique population that frequently have life limiting conditions combined with several comorbidities, cognitive deficits, and frailty. These are associated with a low quality of life and high levels of dependency [15–17]. In addition, mortality rates of nursing home residents are high. Vossius et al. [18] found that approximately 33% of nursing home residents die every year. Similarly, Li et al. [19] reported that 35% of residents die within their first year of admission to the nursing home. This positions nursing homes as a key setting for the provision of high-quality palliative care. However, findings from the literature suggest that the quality of palliative care in nursing homes is unsatisfactory [20]. This may be related to factors such as a lack of staff, training, services, and symptom assessment and management tools [21]. Strategies to improve palliative care provision focus on training staff, initiating advance care planning, and establishing a palliative care team [22]. Additionally, identifying residents with the greatest palliative care needs has been repeatedly advised [22,23].

Screening tools are used in various contexts to identify the presence or risk of a specific disease or problem. Regarding unmet palliative care needs, screening tools use indicators such as pain, depression, and reduced appetite to identify individuals with unmet needs [24,25]. Screening tools have been explored in systematic reviews in other settings for example, in the hospital, emergency department, and primary care [8,9,26–29]. The overall consensus from these reviews is there is no gold standard screening tool recommended for widescale use within these settings. In healthcare, a gold standard refers to widespread acceptance that a method is the “best available” [30].

Only one widely established review exists on screening tools that identify unmet palliative care needs of nursing home residents [31]. Cole et al. [31] evaluated four tools using the COnsensus-based on the Standards for the selection of health Measurement INstruments (COSMIN) criteria [32]. Similar to the other settings, this systematic review determined there was no “gold standard” screening tool being used in nursing homes [31]. While our scoping review also determined the available screening tools, it differs from the review by Cole et al. [31] as there was an additional focus on other methods used to identify unmet needs, specific indicators, referral pathways, and relevant guidelines and frameworks.

Therefore, the aim of this scoping review was to map and summarise the literature on identifying unmet palliative care needs of nursing home residents. The development of the key concepts and research questions were guided by the population, concept, and context framework [33]. The four review questions were as follows:

1. What methods are used to identify unmet palliative care needs of residents in nursing homes?
2. What screening tools have been developed and used to identify unmet palliative care needs of nursing home residents?
3. What guidelines, policies, or frameworks exist regarding identifying unmet palliative care needs of nursing home residents?
4. What indicators are associated with unmet palliative care needs of nursing home residents?

## Methods

### Design

The conduct of this scoping review was guided by the JBI Manual for Evidence Synthesis [33]. The Preferred Reporting Items for Systematic Reviews and Meta-Analyses (PRISMA) extension for scoping reviews [34] was used to guide the reporting of this review (see S1 Checklist). The protocol of this scoping review is registered in Open Science Framework (https://osf.io/3pf8e) and published elsewhere [35].

### Eligibility criteria

The eligibility criteria were based on the population, concept, and context framework [33]. The eligibility criteria are as follows: Population: Residents of a nursing home of any age. Concept: The concept of interest was unmet palliative care needs, as defined in the introduction. Context: Nursing homes. Defined as facilities that provide long term care to residents 24 hours a day. Residents may differ in their level of dependency and require varying levels of assistance with their activities of daily living [36]. Papers that reported on several settings or populations were only included if data from nursing home residents were reported separately.

To reduce the risk of study selection bias, papers with all types of methodologies were considered for inclusion. Grey literature was also considered to ensure a comprehensive representation of the available literature.

As for exclusion criteria, papers reporting solely on symptom assessment of individuals already receiving palliative care were excluded. Tools for identifying acute clinical deterioration were also excluded as this was not the focus for this review [37]. Prognostication-focused papers were excluded as they place an emphasis on predicting life expectancy as opposed to identifying unmet palliative care needs [38]. Papers reporting on the last seven days of life were excluded as the focus of this review was on timely identification of unmet palliative care needs rather than end-of-life symptom experience. Abstracts and protocols were excluded as they did not have enough data for extraction. Studies conducted in any other setting were also excluded. There was no limit placed on date, country, or healthcare system.

### Information sources and search

Following the JBI Manual for Evidence Synthesis, a three-step search strategy was used [33].

The first step involved the search of two databases (Cumulative Index of Nursing and Allied Health Literature [CINAHL] and MEDLINE) using synonyms of keywords (see S1 Search Strategy for full list of synonyms). This initial search was used to identify further synonyms and terms used in the literature. The three search concepts for this review were: palliative care, nursing homes, and screening/identifying needs. Relevant subject headings were identified for each database. Boolean operators ‘AND’ and ‘OR’ were used to refine the search as well as proximity searching. A pilot of the search strategy and eligibility criteria was conducted on 25 random titles and abstracts by PC and NC before implementing the finalised search. There was 100% agreement on the chosen sample.

The second step was the finalised systematic search of all databases: CINAHL, MEDLINE, Embase, Web of Science, APA PsycINFO, and APA PsycArticles. A search of the grey literature was also conducted to ensure the search was comprehensive. The searched grey literature databases were: CareSearch, Trip, GuidelineCentral, and Guidelines International Network. Additionally, ClinicalTrials.gov and the National Institute for Health and Care Excellence website were searched.

The third and final step involved hand searching the reference lists of all included papers to discover additional relevant papers for consideration. Zotero software [39] was used for citation management.

The literature search took place over two months (February and March 2024). See S1 Search Strategy for a complete record of the search strategy for each database.

### Selection of sources of evidence

All titles and abstracts from the bibliographic databases were uploaded to Covidence software [40] and duplicates were removed automatically. Title and abstract screening from the bibliographic databases was conducted with two independent reviewers. To increase the feasibility of this review, titles and abstracts of the grey literature were reviewed by one author (PC) and relevant full texts were uploaded to Covidence thereafter. After title and abstract screening, all papers satisfying the eligibility criteria were sought for retrieval. Only three records by the same author could not be retrieved. The authors sought these records through a university library and by directly contacting the author to no avail.

Two records were in the German language and the DeepL translation tool [41] was used to translate to English. All full text records were uploaded to Covidence, and independent screening was conducted. Any conflicts at any stage during the screening were resolved by a reviewer who was independent of the conflict.

### Data charting and items

Data extraction forms were pre-established and broadly based on the template in the JBI Manual for Evidence Synthesis [33]. These initial forms required adaptation given the heterogeneity of the included papers. The final headings used were: reference, country, time frame, design, aim, sample, resident demographics, method/format, guideline name, purpose, specificity to disease, indicators, assessor/intended user, recommended frequency of assessment, time needed to complete, scoring system, and referral pathway (see S1 and S2 Tables for full details). One reviewer (PC) conducted data extraction. Independent cross-checking was conducted by two reviewers (MMS and NC) on 10% of included papers. Discrepancies were discussed among the reviewers.

### Synthesis of results

Data synthesis was guided by the JBI Manual for Evidence Synthesis [33] as well as Pollock et al. [42]. The characteristics of the included papers were presented including frequencies of the methodologies, countries of origin, language, year, specificity to disease, and sample characteristics. The methods of identifying unmet palliative care needs (including screening tools), guidelines, and referral pathways were synthesised and presented using frequencies, tables, and a visual representation. Basic content analysis was conducted on the relevant indicators [42], which were categorised according to the core domains of palliative care [1,3] and select domains (chosen by relevancy) by Goni-Fuste et al. [4]. Due to the large volume of indicators, the review team added sub-domains to facilitate further organisation and logical presentation. The final categorisation scheme was agreed upon by the review team.

## Results

### Selection of sources of evidence

**Fig 1:**
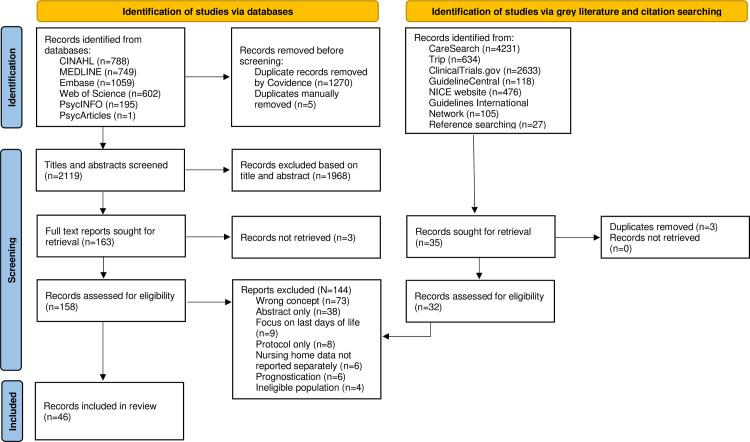
Study identification, screening, and selection process using the PRISMA flow diagram.

The search yielded 3394 results from the bibliographic databases and 8092 results from the grey literature databases. After deduplication of 1275 records, the title and abstracts were screened, resulting in 163 full text records being considered for inclusion. Reference searching of included papers was conducted and 27 further records were considered. A total of 144 records were deemed ineligible during full-text screening. The most common reasons for exclusion were wrong concept (n = 74) and abstract only (n = 38). A total of 46 papers were included in this scoping review. The complete study selection and screening process is presented in Fig 1 [43].

### Characteristics of included papers

Over 60% of the included papers were from the United States of America (n = 16, 35%) and Australia (n = 13, 28%) with the remainder coming from Europe (n = 13, 28%), Canada (n = 3, 6.5%), and New Zealand (n = 1, 2.2%).

All but one paper [44] were published in English. Year of publication ranged from 2002 [45] to 2023 [31,44,46]. There were a total of 36 primary research studies (78%). The remaining records (n = 10, 22%) included a mixture of literature reviews, guidelines, and narrative/discussion papers.

Specificity to disease varied, whereby 70% of the included papers (n = 32) were non-disease specific. However, four of these papers mentioned specific indicators for certain diseases such as dementia, neurological, and cardiovascular disease [31,47–49]. The remaining 14 papers (30%) were focused on dementia, cancer, organ failure, frailty, intellectual disability, or a combination of diseases.

The majority of papers (n = 19, 41%) focused on the residents last six months of life or less. Following that, nine papers (20%) focused on the residents last two years to six months. A further 12 papers (26%) did not specify an exact time frame and five papers (11%) reported an unclear time frame such as ‘final phase’ or ‘near future’ [44,47,50–52]. One paper (2%) had a larger time frame whereby the authors retrospectively analysed data from the Resident Classification Scale over a period of 11 years, from 1997-2008 to identify a trajectory of decline [52].

It is worth mentioning that of the included papers, 21 (46%) collected data retrospectively on the resident’s last months of life. Whereas 13 (28%) collected data prospectively on residents predicted to die. This prediction was most often based on the Surprise Question using time frames ranging from the “near future” [51,52] to 12 months [31,46,47,49]. The Surprise Question involves a healthcare professional asking, “would you be surprised if this person died within X months?”. It was first proposed by Lynn et al. [53] and was intended to prompt referral for palliative or hospice care.

The number of resident participants ranged from 40 [54] to 2730 [55]. Female participants were more frequently represented within the samples, ranging from 52.1-79.7% of the total sample.

Mean age of residents ranged from 72.9 years [56] to 89.1 years [57] while their mean age at death ranged from 72.2 years [58] to 89.9 years [57]. Mean length of residence before death ranged from 4 months [59] to 3.5 years [60].

Residents with dementia participated in 26 of the studies (57%), making it the most frequently represented disease. Participants with dementia ranged from 2% [61] to 100% [50,54–56,62,63] of the total sample. Other common diseases included cancer, heart failure, cardiovascular disease, and chronic pulmonary disease. A summary of some of the study characteristics are presented in Table 1.

**Table 1:** Characteristics of the studies included in the scoping review (n = 46).

Characteristic		n^a^ (%)
**Methodology**		
	Quantitative	22 (47.8%)
	Qualitative	8 (17.4%)
	Mixed methods	6 (13%)
	Narrative/descriptive paper	3 (6.5%)
	Literature review/expert opinion	2 (4.3%)
	Guideline	2 (4.3%)
	Systematic review	1 (2.2%)
	PhD dissertation	1 (2.2%)
	Interview	1 (2.2%)
**Country of origin**		
	United States of America	16 (35%)
	Australia	13 (28%)
	United Kingdom	6 (13%)
	Canada	3 (6.5%)
	Germany	2 (4.3%)
	Spain	2 (4.3%)
	Belgium	2 (4.3%)
	Switzerland	1 (2.2%)
	New Zealand	1 (2.2%)
**Specificity to disease**		
	Non-disease specific	32 (70%)
	Dementia	6 (13%)
	Dementia and non-dementia	4 (9%)
	Dementia and cancer	1 (2.2%)
	Non-cancer	1 (2.2%)
	Cancer/organ failure/frailty/ other	1 (2.2%)
	Intellectual disability	1 (2.2%)

n^a^ = 46 papers

The main outcomes of the included papers varied considerably. Around a third of the papers (n = 14, 30%) focused on improvement of overall palliative care provision and quality of palliative or end-of-life care. The next highest reported outcomes were to identify unmet palliative care needs or eligibility for palliative or end-of-life care (n = 8, 17%) and to identify or describe resident’s needs, symptoms, characteristics, or experiences in their last month to 12 months of life (n = 8, 17%). The remaining 16 papers (35%) had a range of outcomes such as increasing hospice referrals, reducing acute transfers, and increasing advance care planning. Although the scope of the included papers were broad, they were deemed to encompass the concept of identifying unmet palliative care needs in the nursing home population.

### Identifying unmet palliative care needs

In this review, we identified 20 methods or criteria, five screening tools, and four guidelines for identifying unmet palliative care needs of nursing home residents.

### Methods and criteria

There were 20 methods or criteria other than palliative care screening tools identified in 32 papers (70%), with one source identifying five different methods [64]. Most frequently (n = 6, 19%), a unique criterion [65–67] or assessment [63,68,69] was reported. These assessments included combinations of multiple tools such as the Cognitive Performance Scale, the Mini Mental State Exam, and the Geriatric Depression Scale, full details are outlined in S1 Table. The most frequently reported method (n = 5, 16%) was the Minimum Data Set (MDS). Two of which used the Resident Assessment Instrument(RAI)-MDS [60,70], and one used a combination of both the MDS and RAI-MDS [57]. Furthermore, three additional papers [57,68,70] reported the use of the RAI-MDS as one component of the assessment. Five papers (16%) identified a method using staff or relative interviews/questionnaires, group discussions, or a combination thereof [45,50,58,64,71]. Three papers (9%) reported the use of staff or expert opinion [44,54,64]. A further three papers (9%) did not outline the specific method or criteria used [57,72,73]. All three corresponding authors were contacted for further details, but no response was received.

Palliative care tools such as the Palliative Care Outcome Scale, the Palliative Care Outcomes Collaboration tools, and the Palliative Performance Scale were included as part of some methods. These tools were developed as core outcome measures for advanced cancer patients [74], and specialist palliative care [75], and to measure the physical condition of palliative care patients [76]. While they have been used as part of a method in the included studies, they were not developed as an independent screening tool and were therefore not categorised as such. The identified methods are summarised in Table 2.

**Table 2:** Methods or criteria used to identify unmet palliative care needs within the studies included in the scoping review.

Method or criteria	n^a^ (%)
Unique criteria or assessment [63,65–69]	6 (19%)
MDS (including RAI-MDS) [55,59–61,64]	5 (16%)
Staff or relative interviews/questionnaires, group discussions, or a combination [45,50,58,64,71]	5 (16%)
Staff/expert opinion [44,54,64]	3 (9%)
Criteria not outlined [57,72,73]	3 (9%)
Nursing notes or chart review [62,64]	3 (9%)
Palliative Care Needs Rounds [77,78]	2 (6%)
Gold Standards Framework [79,80]	2 (6%)
Chart review and MDS [70]	1 (3%)
Resident interview [64]	1 (3%)
POS and SQ [81]	1 (3%)
CFS and PPS [82]	1 (3%)
SECPAL criteria, ESAS, and POS [83]	1 (3%)
PCOC criteria [84]	1 (3%)
InterRAI [85]	1 (3%)
Resident Classification Scale^*^ [86]	1 (3%)

**CFS:** Clinical Frailty Scale; **ESAS:** Edmonton Symptom Assessment Scale; **InterRAI;** International Resident Assessment Instrument; **MDS:** Minimum Data Set; **PCOC:** Palliative Care Outcomes Collaboration; **POS:** Palliative Outcome Scale; **PPS:** Palliative Performance Scale; **RAI:** Resident Assessment Instrument; **SECPAL:** Spanish Society of Palliative Care; **SQ:** Surprise Question.

n^a^ = 32 papers.

* After March 2008, assessments changed from the Resident Classification Scale to the Aged Care Funding Instrument in Australia.

It should be mentioned that 15 (47%) of these papers identified a method or criteria to facilitate identifying unmet palliative care needs. Whereas 12 (38%) used various methods to describe residents’ needs, symptoms, indicators, or characteristics. The remaining three (9%) reported both methods and descriptions.

Furthermore, only three papers (9%) focused on what methods are used by healthcare professionals in daily practice [44,54,71] rather than a method implemented as part of an interventional study.

### Screening tools

The identified screening tools varied and were aimed at (1) identifying palliative care needs [31,47,49], (2) identifying palliative care needs and prompting further assessment [46,87–90], (3) identifying symptoms and prompting palliative care referral [56], and (4) prompting palliative care planning [54].

There were five screening tools reported in ten papers, all of which were paper based. The Palliative Care Needs Rounds (PCNR) Checklist was the most reported tool (n = 4) [87–90]. Followed by the Necesidades Paliativas (NECPAL) tool which translates to “Palliative Needs” and was reported in one study [47] and one systematic review [31]. The Supportive Palliative Care Indicators Tool (SPICT) in combination with the Surprise Question was reported in one paper [49]. The SPICT was also mentioned in another paper [46] and was used in combination with the Surprise Question and the Australian Modified Karnofsky Performance Scale. The Palliative Care Screening for the Elderly tool was reported in one paper [56] and a modified Gold Standards Framework-Proactive Indicator Guidance [54] was also reported in one paper. The identified screening tools are summarised in Table 3.

**Table 3:** Screening tools used in the included studies to help identify unmet palliative care needs.

Screening tool	n^a^(%)
PCNR Checklist [87–90]	4 (40%)
NECPAL [31,47]	2 (20%)
Palliative Care Screening for the Elderly tool [56]	1 (10%)
SPICT and SQ [49]	1 (10%)
SPICT, SQ, and AKPS [46]	1 (10%)
Modified GSF-PIG [54]	1 (10%)

AKPS Australian Modified Karnofsky Performance Scale; **GSF-PIG:** Gold Standards Framework- Proactive Identification Guidance; **NECPAL:** Necesidades Paliativas (Palliative Needs); **PCNR:** Palliative Care Needs Rounds; **SPICT:** Supportive and Palliative Care Indicators Tool; **SQ:** Surprise Question

n^a^= 10 papers

### Guidelines

There were no clinical guidelines, frameworks, or policies found in the search relevant to identifying unmet palliative care needs of nursing home residents. Four guidelines were identified from seven sources.

One guideline identified was developed by the National Health Service in the United Kingdom and was reported in two sources [51,52]. This guideline is part of the Department of Health’s End of Life Care Strategy [91] and is aimed at improving the quality of end-of-life care in care homes. It outlines a six step care pathway beginning with identifying triggers to initiate end-of-life planning and ending with care after death.

Three papers reported the use of the National Hospice Organization [65,69] or adapted National Hospice Organization guidelines [48] which were developed in the United States of America. These guidelines were used to determine palliative care eligibility for residents without cancer [48], to determine hospice eligibility [69] and they were used after palliative care screening to inform further assessments [65].

Another guideline was identified titled “Guidelines for a Palliative Approach in Residential Aged Care” [92] and was developed in Australia and supplied to all residential aged care facilities in the country. This is a broad guideline providing extensive advice on palliative care delivery.

The last guideline was developed in Canada and titled “Nursing Guidelines for End-of-Life care in Long-Term Care Settings” [93]. It is aimed at prompting nurses in the commencement of end-of-life care. This guideline was the only non-disease specific guideline that outlines a composite measure and associated recommendations. The scoring system is based on the Palliative Performance Scale and interventions are recommended based on the recorded score. For example, a resident with a score of 40% would have recommendations such as conducting frequent reviews and educating the resident and their relatives on the possible deterioration of their health.

### Indicators of unmet palliative care needs

The included papers had high heterogeneity and therefore used different terminology such as characteristics, needs, symptoms, predictors, or factors to describe unmet palliative care needs. These have all been broadly considered under the general term of indicators. An indicator can be defined as something that demonstrates the presence or degree of a problem [94]. Nineteen papers (41%) outlined a composite measure for screening [31,46–49,54,56,65–67,77,78,82,83,87–90,93]. These composite measures included multiple indicators and sometimes outlined a specific score for each indicator such as Body Mass Index < 20 for example [49]. However, all other indicators were individually extrapolated from a combination of sources as described above. These papers were often less specific and simply reported the presence of an indicator.

There were 117 different indicators reported and they have been categorised into the core domains of palliative care [1,3] and the relevant domains of needs assessments in palliative care by Goni-Fuste et al. [4]. The domains used were as follows: physical (n = 58, 49%), psychological (n = 22, 19%), social (n = 2, 2%), spiritual (n = 3, 3%), information (n = 1, 1%), practical (n = 5, 4%), autonomy (n = 1, 1%), personal issues (n = 1, 1%), and healthcare (n = 24, 20%).

**Fig 2:**
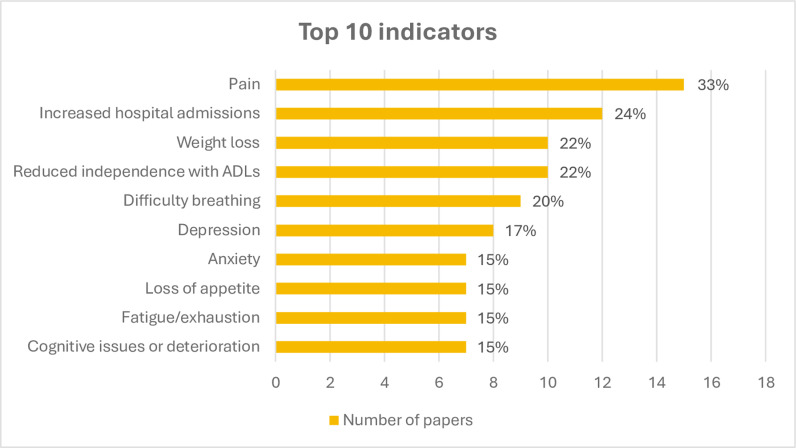
Top ten reported non-disease specific indicators of unmet palliative care needs in the included studies. ADL: Activities of Daily Living.

Indicators were separated into non-disease specific and disease specific. Non-disease specific indicators were reported in 35 papers (76%) and the full list is outlined in Table 4. The core domains and the top ten reported indicators are summarised hereafter. Complete details of both the disease specific and non-disease specific indicators are provided in S1 and S2 Tables. The top ten reported non-disease specific indicators are illustrated in Fig 2.

**Table 4:** Non-disease specific indicators of unmet palliative care needs.

Domains	Indicators		
**Physical** (N = 57)	**Hydration and nutrition** Weight loss (n = 10) Loss of appetite (n = 8) Nausea (n = 6) Impaired nutrition (n = 4) Reduced intake of fluids (n = 4) Vomiting (n = 2) Difficulty eating (n = 2) Low Body Mass Index (n = 1) Cachexia (n = 1) Digestive dysfunction (n = 1) Requires assistance with eating (n = 1) **Oral** Dysphagia (n = 3) Choking (n = 2) Xerostomia (n = 2) Oral problems (n = 1) Mucositis (n = 1) **Acute events** Acute event/illness (n = 2) Recurring infections (n = 1) Urinary Tract Infection (n = 1) Pneumonia (n = 1) Crisis requiring discussion about end of life (n = 1)	Feeling unwell (n = 1) **Skin integrity** Pressure ulcers (n = 5) Impaired skin integrity (n = 1) Rash (n = 1) Oedema (n = 1) **Excretion** Constipation (n = 5) Incontinence (n = 4) Difficulty urinating (n = 1) Diarrhoea (n = 1) **Mobility and strength** Reduced mobility (n = 4) Increased falls (n = 2) Frailty (n = 2) Reduced activity (n = 1) Reduced handgrip (n = 1) Reduced strength (n = 1) **Respiratory** Difficulty breathing (n = 9) Cough (n = 2) Altered breathing (n = 1) Oxygen therapy (n = 1) Ventilated (n = 1) Noisy respirations (n = 1)	**Disease and deterioration** Disease progression/deterioration (n = 6) Persistent or worsening symptoms (n = 5) End-stage/life-limiting/advanced disease (n = 3) Comorbidities (including Charlson Comorbidity Index) (n = 3) Unstable condition (n = 2) Physical deterioration (n = 1*) Diagnosis (n = 1) Health instability (CHESS) (n = 1) **Fatigue and sleep** Fatigue/exhaustion (n = 7) Difficulty sleeping (n = 5) Weakness (n = 3) Restlessness (n = 1) Other Pain (n = 15) Geriatric syndromesa (n = 1) ‘Other’ physical symptom (n = 1)
**Healthcare** (N = 24)	**Treatment** Chemotherapy (n = 1) Dialysis (n = 1) Radiation (n = 1) Parenteral therapy (n = 1) Intravenous therapy (n = 1) Low expectation to respond to treatment (n = 1) Had CPR performed (n = 1) Quantity of medications (n = 1) **Care** Increased hospital admissions (n = 12) Direct transfer for end-of-life care (n = 1*) Requires 24 hour nursing care (n = 1) Use of healthcare resources (n = 1)	Requires more nursing care (n = 1) **Staff and relatives** Staff identify need for palliative care (n = 2) Disagreements between staff and relatives about treatment and plan of care (n = 1*) **Advance Care Planning** Lack of Advance Care Planning (n = 2*) No care plan for the last 6 months of life (n = 1*) **Advance Care Directive** Do Not Resuscitate (n = 4) Do Not Hospitalise order (n = 2)	Orders to stop other treatments (n = 1) Documentation of refusal for mechanical ventilation (n = 1) **Prognosis** Prognosis of less than 6 months (n = 3) Death anticipated (n = 1) **Positive Surprise Question** Near future (n = 2) 1 week to 2 weeks (n = 1) 15-28 days (n = 1) More than 28 days (n = 1) 6 months (n = 4*) 6 to 12 months (n = 1) 12 months^d^ (n = 4)
**Psychological** (N = 22)	Depression (n = 8) Anxiety (n = 7) Cognitive issues or deterioration (including CPS and Pfeifferc) (n = 7*) Delirium (n = 5) Reduced level of consciousness (n = 3) Drowsiness (n = 3) Sadness (n = 2)	Loneliness (n = 2) Requires emotional support or preparation (n = 2) Worry (n = 2) Nervousness (n = 2) Emotional distress (n = 2*) Challenging behaviour (n = 1) Severe Adaptive Disorderb (n = 1) Confusion (n = 1)	Emotional impact on resident (n = 1) Need for counselling or psychological care (n = 1) Difficulty concentrating (n = 1) Aggression (n = 1) Lacking self-worth (n = 1) Difficulty expressing feelings (n = 1) Having a desire to live (n = 1)
**Practical** (N = 5)	Reducing independence with ADLs (including Barthel Index) (n = 10) Functional deterioration (including Karnofsky Performance Scale) (n = 6)	Requires help with personal hygiene (n = 6) Increased dependency (n = 4)	Requires help with dressing (n = 1)
**Spiritual** (N = 3)	Requires more support spiritually (n = 3)	Engage in spiritual practices (n = 1)	Expression of spirituality (n = 1)
**Social** (N = 2)	Social withdrawal or isolation (n = 2*)	Social needs (n = 1)	
**Personal issues** (N = 2)	Personal matters (e.g., financial) (n = 1)	Significant life event (e.g., death of spouse) (n = 1)	
**Information** (N = 1)	Needs more information (n = 1)		
**Autonomy **(N = 1)	Resident or relative choosing palliative care (n = 5*)		

**ADL:** Activity of Daily Living; **CHESS:** Changes in Health, End-Stage Disease and Signs and Symptoms; **CPR:** Cardiopulmonary Resuscitation; **CPS:** Cognitive Performance Scale; **SQ:** Surprise Question.

N =  total number of indicators in domain.

n =  number of studies the indicator was reported in.

*Indicator reported in the same tool/guideline from multiple sources and frequency reduced accordingly.

a Includes a range of conditions such as: dementia, frailty, depression, delirium, vertigo, incontinence.

b Excessive reactions to stress.

c Test for cognitive deficiency.

d One paper recommends replacing with the Frailty VIG Index.

### Physical domain

The physical domain had the largest number of indicators (n = 57, 48%). Pain was reported in 15 papers (33%) and was the highest reported indicator overall. Some papers reported either the presence or absence of pain rather than using a specific tool or score. There was a variety of methods used for measuring pain such as the RAI-MDS [60,68], the Pain Index Scale [57], the Abbey Pain Scale and the Verbal Descriptor Scale [48], the Palliative Outcome Scale [81], the Edmonton Symptom Assessment Scale [83] and the Memorial Symptom Assessment Scale [67,69]. Other papers reported frequencies and odd ratios for the presence of daily pain [60,68], moderate to severe pain [48,61], or severe to excruciating pain [61].

Weight loss (n = 10, 22%) was the second highest reported indicator within the physical domain. Some papers reported frequencies of any weight loss, while others identified specific thresholds. Liyanage et al. [49] reported 5-10% weight loss in the last 3-6 months. Similarly, Grbich et al. [48] and Cole et al. [31] reported weight loss of more than 10% in the last 6 months.

Difficulty breathing (n = 9, 20%) was the next highest reported physical indicator, followed by loss of appetite and fatigue/exhaustion which were reported in seven papers (15%) each.

### Psychological domain

The psychological domain had 22 indicators (19%) overall with depression being the highest reported indicator (n = 8, 22%) in this domain. Depression was measured using the MDS-Depression Rating Scale [57,60,61], the Geriatric Depression Scale [69], the Edmonton Symptom Assessment Scale [83], and through staff interview [45]. Other papers did not mention the method or simply used the presence or absence of depression. Some papers reported frequencies and odds ratios of depression [45,48,61,66,83]. After depression, anxiety and cognitive issues or deterioration were reported in seven papers each (15%) and were the next highest reported indicators within the psychological domain.

### Social domain

The social domain had just two indicators (2%). Social withdrawal or isolation (n = 3, 6.5%) was the highest reported indicator within this domain. Two methods of identification were reported: informal staff recognition [44], and the NECPAL [31,47].

### Spiritual domain

The spiritual domain had three indicators (3%). Requiring more support spiritually (n = 3, 6.5%) was the highest reported indicator within this domain. One paper reported the use of staff interviews [45], while the other two did not specify an exact method [66,67].

### Healthcare domain

The healthcare domain had the second highest number of indicators (n = 24, 20%). Increased hospital admissions (n = 12, 26%) was the highest reported indicator in this domain and the second highest overall. Two papers were specific, stating two or more hospitalisations within the previous six months [31,49]. Whereas the other papers simply reported more frequent or recurrent hospital admissions.

A positive response to the Surprise Question was mentioned as an indicator in 11 papers (24%). However, it was not included in the top ten indicators due to the variation in time frames used. The Surprise Question was only included in this review when the focus was on identifying unmet palliative care needs rather than prognostication. Six papers [31,46–49,81] used the Surprise Question as an essential criterion for palliative care eligibility. Although Esteban-Burgos et al. [47] recommended the replacement of the Surprise Question with the Frailty VIG (Spanish abbreviation for Comprehensive Geriatric Assessment) Index.

### Practical domain

The practical domain had five indicators (4%). The highest reported indicator (n = 10, 22%) in the practical domain was reducing independence with activities of daily living (including the Barthel Index). One paper [31] outlined a precise score of < 20 for the Barthel Index, where a lower score indicates a higher dependency (score of 0-20 indicates complete dependency). Likewise, Grbich et al. [48] reported a mean Barthel Index of 2.3. Whereas Estabrooks et al. [60] reported that 82% of residents had a MDS Activity of Daily Living score of 15 + , where a higher score indicates higher dependency (maximum score of 28). Similarly, Lima and Miller [68] reported a mean MDS Activity of Daily Living score of between 17.27 and 17.69 for residents who received a palliative care consult. All of which signifying residents’ decreasing independence with activities of daily living towards end of life.

### Disease specific indicators

Indicators specific to certain diseases were also identified. While these indicators were often the same as the non-disease specific, some diseases had unique indicators. Only the indicators distinct from the non-disease specific indicators are reported below. However, S1 and S2 Tables provide a complete list of both sets of indicators.

The most frequently reported disease was dementia, dementia specific indicators include: pyrexia [59,71], pyelonephritis [54], aspiration pneumonia [54], an increase in antipsychotics [60], agitation [62,63], lack of interest [62], hallucinations [59], fear [63], overstimulation (e.g., too much light) [50], need for stimulation (e.g., sensory interventions to promote wellbeing) [50], convey emotions [50], meaningful oral communication [54], being understood/conveying their wishes [50], experiencing daily activities [50], being themselves [50], opposing care [63], having familiar surroundings [50], and safety [50]. Liyanage et al. [49] reported dementia and frailty combined indicators which include: femoral fracture, pyrexia, aspiration pneumonia, and inability to communicate.

Cancer specific indicators include: pyrexia, and hallucinations [59]. Neurological disease specific indicators include: aspiration pneumonia, respiratory failure, and consistent decline in cognitive functioning regardless of treatment [49]. Cardiovascular disease specific indicators include: New York Heart Association stage III/IV heart failure, coronary artery disease, and severe peripheral vascular disease [49]. Respiratory disease specific indicators include: chronic obstructive pulmonary disease, pulmonary fibrosis, and required ventilation as a result of respiratory failure [49]. Kidney disease specific indicators include: chronic kidney disease, kidney failure, and discontinuing dialysis [49]. Liver disease specific indicators include: liver cirrhosis, contraindicated for a liver transplant, and one or more clinical indicator (no further elaboration provided) [49]. Intellectual disability, organ failure, and frailty also had specific indicators however they were not unique from the non-disease specific indicators in Table 4.

### Palliative care screening characteristics

The included methods, screening tools, and guidelines presented information on several aspects of the palliative care screening and assessment process. This included frequency of assessment, time to complete assessment, and the relevant assessor.

Frequency of assessment ranged from daily (if the resident was deteriorating) [72] to annually [54,55,61,86]. Length of time the assessment took ranged from 18.6 seconds [49] to 1.5 hours [69], with one paper stating ‘minimal time’ [56]. Each method had an individualised scoring system which are detailed in S1 and S2 Tables. Assessments were primarily conducted by nursing home staff including directors of nursing, managers, team leaders, registered nurses, nurse assistants/healthcare assistants, activity coordinators, nursing administration, and cleaners. Other professionals included specialists in palliative care, general practitioners/consultants, advanced nurse practitioners, psychologists, dietitians, social workers, speech and language therapists, and chaplains. On four occasions the researcher conducted the assessment [62,67,69,82]. Less frequently, relatives [50,64,71] were involved in the assessment and only one paper [64] identified residents’ involvement through the use of interviews. However, it should be noted that seven papers (15%) reported an indicator for residents or relatives identifying the need for palliative care [31,45,47–49,67,92] as described in Table 4.

### Referral pathways

**Fig 3:**
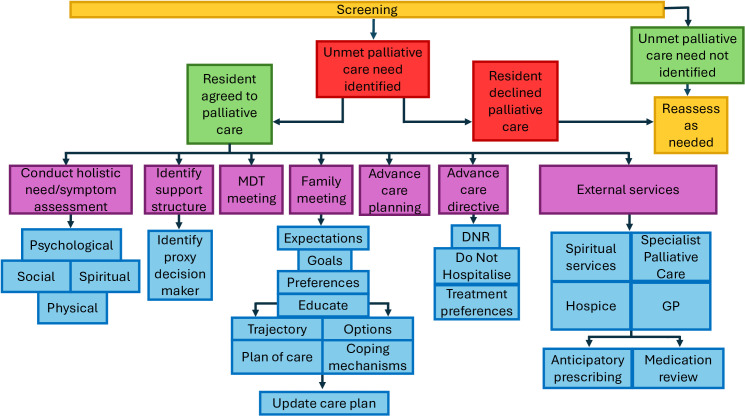
Palliative care pathway based on most frequently reported interventions in the included studies.

Five papers reported an explicit referral or care pathway [44,51,67,88,93]. It is worth mentioning that the NECPAL [95] and the GSF-PIG [96] have their own pathway, which was not reported in the included papers. Both Pruitt [56] and Robinson et al. [85] mentioned referrals or interventions however no further elaboration was provided. All remaining papers identified appropriate interventions or actions to be taken in a less formal capacity. Fig 3 provides a summary visual presentation of a palliative care pathway based on the most frequently reported interventions and actions from all of the included papers.

## Discussion

This scoping review aimed to map and summarise the literature on identifying unmet palliative care needs of nursing home residents. While there was a review evaluating relevant screening tools [31], other methods, relevant indicators, pathways, and guidelines were unmapped prior to the current scoping review. From the 46 included records, we identified 20 methods, five screening tools, four guidelines, and 117 indicators to identify unmet palliative care needs of nursing home residents.

The concept of ‘unmet palliative care needs’ is mentioned repeatedly in other settings [9,26] as well as specific to the nursing home [31,49,83]. However, it is lacking a clear definition. What is clear is that this concept is multi-faceted and this is evident through the variety of outcomes used in the included studies. These include: to improve palliative care provision, to increase palliative care or hospice referrals, to increase advance care planning, and to reduce acute care transfers. Frequently, determining eligibility for palliative care was used in combination or interchangeably with the concept of identifying unmet palliative care needs. Distinguishing between these two concepts is a challenge due to their similarities. Therefore, the concept of ‘unmet palliative care needs’ would benefit from a conclusive theoretical and empirical definition facilitating greater understanding and accurate measurement in nursing home residents [97].

The residents in the included papers shared some similar characteristics. In particular, males and those with non-dementia conditions were less represented. However, this was not unexpected given the high numbers of females [98,99] and the prevalence of dementia in nursing homes [100].

### Methods of identifying unmet palliative care needs

Methods other than screening tools to identify nursing home residents unmet palliative care needs were diverse. Twenty unique methods were reported. While developing and adapting methods according to specific cultures and healthcare systems is sometimes necessary, this large variety may be superfluous and could result in inconsistencies in the identification of these needs.

Standardising methods of identifying unmet palliative care needs of nursing home residents may be of benefit. Standardising methods can lead to consistency, better outcomes, less errors [101], and improved perceptions of care from the healthcare user [102]. Standardised approaches also allow for comparison of care and health outcomes across different healthcare settings and systems. A standardised method of identifying nursing home residents’ unmet palliative care needs could support consistency of palliative care delivery across different facilities and countries. However, evidence is needed to support the advocacy of a ‘gold standard’ method. Objective evaluations such as the COSMIN criteria are useful in providing a critical analysis and recommendations of available instruments [31,32]. COSMIN should be used to guide further development of instruments for nursing home residents to ensure a valid and reliable tool is developed.

However, consideration should be given to allow for customisation of standardised methods to provide flexibility to adapt to individual circumstances and needs [103]. Furthermore, there may be a need to culturally adapt methods in different regions and healthcare systems. For example, items may not translate effectively into another language. Therefore, cross-cultural adaptation and validation of tools used outside of the culture or language in which it was originally developed should be conducted [104]. In addition, infrastructure across healthcare systems may vary. For example, some countries such as Belgium and Denmark [105] are quite advanced in terms of their use of digital and eHealth. Whereas other countries such as Ireland and Romania are in the early stages of development in comparison to the rest of Europe [105]. As a result, an electronic tool may work effectively in one country and not another and paper-based adaptations may be necessary.

Our findings demonstrated that residents and their relatives were not often involved in the assessment. A qualitative study in four Norwegian nursing homes found that residents and their relatives valued being involved in advance care planning [106]. It contributed to trust between the nursing home staff and residents and to a more person-centred approach to their care. Furthermore, shared decision-making whereby the patient is a part of healthcare decisions is increasingly encouraged among healthcare professionals [107,108]. Timely shared decision-making is of particular importance when discussing end-of-life planning as residents are likely to lose capacity to make decisions as they age and their disease progresses [109]. Accordingly, healthcare professionals should involve residents in assessments of their unmet palliative care needs.

It should be emphasised that the majority of methods included in this review were often implemented as part of an interventional study rather than implemented as part of routine care. Only three papers [44,54,64] concentrated on what methods healthcare professionals use in practice. These methods included staff identification based on knowledge and experience and using a modified GSF-PIG. Two papers were literature reviews [54,64], although one [54] had a quantitative retrospective analysis component. The third paper was a qualitative study [44]. The evidence that palliative care is insufficient in nursing homes is clear [13,110]. This low number of studies on what methods are used by healthcare professionals in daily practice contributes to this insufficiency. Identifying residents with the greatest palliative care needs has been repeatedly advised [22,23]. Consequently, the most effective methods of identification need to be determined. Future research should focus on identifying what methods staff are routinely using in practice. Such research could use surveys or focus groups of nursing home staff. Knowing what, if any, methods nursing home staff are routinely using could facilitate the development of targeted interventions. Alternatively, it may uncover informal methods being used by staff that have potential for adaptation and refinement. Furthermore, research needs to focus on determining the barriers to implementing such methods in nursing homes for example, a lack of staff or resources [21]. This would provide valuable insights to inform future intervention implementation.

### Screening tools

Similar to Cole et al. [31], we identified five screening tools from the literature. However, one tool “The Palliative Care Screening for the Elderly Tool” by Pruitt [56] was not identified in the review by Cole et al. [31] as it was published after their search was conducted. This tool was developed specifically for nursing home residents with dementia and information on its development and testing is limited [56]. The PCNR checklist [88] was also developed for the nursing home setting. However, the absence of data regarding its content validation led to its exclusion from the review by Cole et al. [31]. In addition, the SPICT [49], the NECPAL [47], and the modified GSF-PIG [54] were used in the nursing home setting. However, all three were developed unspecific to a particular setting [96,111,112].

All identified screening tools in this review were paper based. Other settings such as the hospital [113] and primary care [114] have made developments in the use of electronic screening tools. These tools use routine data to identify a person’s appropriateness for palliative care and to identify palliative care needs. Mason et al. [114] developed such a tool for use in the primary care setting. This tool identified 0.8% of patients from eight practices. The participating General Practitioners expressed many of these patients would not have been identified without the tool, particularly those with non-cancer conditions. Electronic tools have yet to be explored within the nursing home setting. Nursing home staff have expressed their difficulty in determining whether a resident is suitable for palliative care [44,115]. Furthermore, their knowledge of palliative care has been shown to be inadequate [115] and disparate [116]. Therefore, electronic tools within nursing homes have potential to support staff’s decision-making and improve the provision of palliative care to residents. However, participants of the study by Mason et al. [114] raised their concern that screening could significantly increase workload. This is an important consideration when developing such a tool to ensure its successful implementation and feasibility.

After we conducted our search, Cole et al. [117] published a palliative care referral criterion. Interviews of 17 nursing home and palliative care professionals informed this criterion. Five distinct themes were identified as well as a range of indicators which are comparable to those identified in this scoping review. Cole et al. [117] intend for their criteria to be the groundwork for the development of a screening tool specific to the nursing home setting and is a valuable addition to this underdeveloped concept.

### Guidelines

No clinical practice guidelines or policies for identifying unmet palliative care needs of nursing home residents were found. Clinical practice guidelines provide evidence-based recommendations typically based on the findings of a systematic review [118]. Clinical practice guidelines can support healthcare professional’s decision-making and provide guidance on best practice. Working towards such a guideline for identifying unmet palliative care needs of nursing home residents may begin with the establishment of a steering group inclusive of relevant stakeholders [119]. However, a steering group should include local and international multidisciplinary members to provide broad perspectives on the topic [119]. A clinical practice guideline would support standardisation and evidence-based practice for staff in nursing homes worldwide. Without clear guidelines, nursing home staff may rely on informal practices that are not evidence-based. In addition, a lack of guidelines creates challenges for auditing practice and identifying areas for improvement.

The National Institute for Health and Care Excellence have developed a clinical guideline titled “End of life care for adults: service delivery” [12]. This guideline provides recommendations on identifying individuals nearing end of life and conducting needs assessments. While it provides beneficial generalised advice on end-of-life care, it is not specific to nursing home residents and was therefore excluded from the current review.

Four guidelines were identified in this review. The “Nursing Guidelines for End-of-Life care in Long-Term Care Settings” [93] was the only non-disease specific guideline that outlined a composite measure and associated interventions. The scoring system is based on the Palliative Performance Scale and interventions are recommended based on the recorded score. The included paper [93] reports on the development and initial evaluation of these guidelines in 35 Canadian long-term care homes. Overall, participants rated the guidelines as “very good” and some participants reported an improvement in the timeliness of end-of-life needs identification. However, Chu et al. [120] propose the Palliative Performance Scale may be most useful for the prediction of those in their last days of life. Therefore, further evaluation is needed to determine these guidelines appropriateness for timely identification of resident’s palliative care needs. Furthermore, staff in one mixed-methods study [121] feared that the Palliative Performance Scale would trigger excessively as many residents receive a low score suggestive of a short life expectancy. This may result in unfeasible workload implications for staff which is an important consideration when implementing such a guideline.

The National Health Service guideline [51,52] and the “Guidelines for a Palliative Approach in Residential Aged Care” [92] provide broad advice in relation to palliative care delivery in the long term care setting. However, neither document outlines a composite measure for identifying unmet palliative care needs or eligibility for palliative care.

The last guideline identified was the National Hospice Organization Guidelines [48,65,69]. These guidelines were originally developed to determine hospice eligibility with specific reference to the Medicare Hospice Benefit in the United States of America [122]. As this guideline concentrates on eligibility for hospice, it’s applicability for use for early identification of unmet palliative care needs is questionable. Furthermore, these guidelines were not developed for nursing home residents, rather for any individual with a noncancer disease.

Considering the available guidelines, a guideline developed specifically for the nursing home setting may be warranted. Such a guideline should include an exact scoring system for identifying residents’ unmet palliative care needs. A clear composite measure would support staff’s decision making in implementing timely palliative care interventions. However, similar to standardisation, implementing a guideline may limit individualisation of care whereby all residents are assessed with the same criteria regardless of unique circumstances and preferences [123]. Caution in the use of rigid guidelines is advised [124] in view of factors such as limited generalisability and risk of bias during development. Accommodations should be made to allow for individualisation of care. This may be achieved by including residents in the guideline development as well as considering individualisation at all stages of development and implementation [123].

### Indicators of unmet palliative care needs

There were 117 different indicators for identifying residents unmet palliative care needs. It should be noted that the NECPAL [95] and the GSF-PIG [96] have additional criteria that were not reported in the included papers and therefore were not included in this review.

Pain was the highest reported indicator overall which is consistent with other literature in nursing homes. Thompson et al. [125] conducted a study using the MDS-RAI evaluating 962 residents’ pain at end of life. Over half of the participants experienced pain in the six months before death with 34.6% experiencing moderate to severe pain and only 5.3% feeling an improvement in their pain before death. Furthermore, Smets et al. [116] reported that nursing home staff in five European countries had large variances in knowledge of pain management at end of life. This reinforces the importance of improving pain assessment and management for nursing home residents at the end of their life. It is worth noting that symptoms such as pain related to palliation are distinct from those associated with acute injury or trauma for example, as a result of a fall.

Physical indicators represented almost half of the total number of indicators included in the current review. Other domains such as social and spiritual were significantly less represented. A white paper by van der Steen et al. [126] emphasised the importance of social and spiritual needs in palliative care for older people with dementia. Similarly, a qualitative study [127] evaluated spirituality in end-of-life care, reporting that it provides patients with a sense of meaning towards end of life. Thus, needs beyond the physical domain should be considered to ensure a comprehensive and holistic assessment of resident’s palliative care needs.

Some of the included papers identified “Do Not Resuscitate” and “Do Not Hospitalise” orders as an indicator of hospice eligibility or were associated with residents at end of life [45,61,67,85]. However, these orders were also frequently part of referral pathways and recommended interventions [46,54,55,64,68,72]. Therefore, it could be argued that those who do not have these anticipatory orders in place but would benefit from it, have an unmet need.

The Surprise Question was mentioned in 11 papers, six of those used it as an essential criterion for palliative care eligibility. However, it’s prognostic ability has been examined in more recent years and it has been criticised for its subjectivity and varied sensitivity and specificity [128]. Nevertheless, the focus of this scoping review is not on prognostication, rather on timely identification of unmet palliative care needs. With this in mind, the Center to Advance Palliative Care [129] state that eligibility for palliative care should be based on an individual’s needs rather than prognosis. Consequently, the Surprise Question as a complimentary rather than essential criterion as part of palliative care screening may be more appropriate. Alternatively, Esteban-Burgos et al. [47] recommend replacing the Surprise Question outright with the Frailty VIG Index as nursing home residents displayed palliative needs irrespective of a positive Surprise Question. The Frailty VIG Index is used to score and determine an individual’s degree of frailty [130] however, it was developed and evaluated in Spanish. Therefore, translation and validation of the Frailty VIG Index is needed in other languages.

## Recommendations

A number of recommendations to guide future research can be made from the findings of this review which include: (1) Defining the concept of ‘unmet palliative care needs’ to facilitate its assessment and measurement in nursing home residents (2) Future studies on what methods nursing home staff use in routine practice to identify unmet palliative care needs. This could guide and inform future interventions to improve nursing home staff’s identification of unmet palliative care needs. (3) A promising area for future research would be in the development of an electronic decision support tool to strengthen nursing home staff’s decision-making of when and how to provide timely palliative care. (4) Residents should be included as part of palliative care needs screening in research as well as in practice. (5) Further evaluation of the “Nursing Guidelines for End-of-Life care in Long-Term Care Settings” is warranted to determine the guidelines effectiveness in timely identification of palliative care needs.

## Limitations

To increase the feasibility of conducting this review, the title and abstract screening of the grey literature was conducted by one reviewer (PC), increasing the risk of reviewer bias. Three full text records could not be retrieved and were therefore excluded from this review without screening. A further limitation may be that risk of bias and quality appraisal of the included papers was not performed. However, this is not typically required in a scoping review [33] and our aim was to scope the overall body of evidence regardless of methodological quality or risk of bias. While we included papers irrespective of language, the use of translation software (DeepL) for two papers had the potential to introduce errors.

## Conclusions

This is the first scoping review to map and summarise the evidence on identifying unmet palliative care needs of nursing home residents. Determining nursing home residents’ eligibility for palliative care and identifying the associated unmet needs is an undeniably complicated task. This review demonstrates that there is significant heterogeneity in the methods, tools, and guidelines used to identify unmet palliative care needs in nursing home residents. Added complexities include the lack of a clear definition of the concept ‘unmet palliative care needs’. Future research should focus on providing a clear definition of this concept as well as working towards evidence-based standardisation within the nursing home context. The overall goal being to ensure timely and equitable access to palliative care for nursing home residents. Achieving this goal requires screening for unmet palliative needs to be incorporated into routine care.

## Supporting information

S1 Checklist PRISMA-ScR checklist. (DOCX)

S1 Search Strategies Database search strategies. (DOCX)

S1 Table Data extraction table 1: Methods of identifying unmet palliative care needs. (DOCX)

S2 Table Data extraction table 2: Guidelines. (DOCX)
