# Supplementary material for: Identifying unmet palliative care needs of nursing home residents: A scoping review
Source: PLoS One. 2025 Feb 25;20(2):e0319403. doi: 10.1371/journal.pone.0319403 (PMC11856323; doi:10.1371/journal.pone.0319403)
Supplement: S1 Search Strategies — (DOCX) [file pone.0319403.s002.docx]

**S1. Search Strategies**

**Table 1. Search concepts and terms**

| **Search concepts** | **Palliative** | **Nursing Homes** | **Screening/Identifying Needs** |
| --- | --- | --- | --- |
| **Search terms** | Palliat*, end of life”, end-of-life, EOL, terminal, terminally, hospice*, dying, “last year of life” | Nursing home*, long term care, long-term care, retirement home(s), residential care, residential home(s), residential facility, residential facilities, assisted living, assisted-living, old-age home(s), homes for the aged, care home(s) | Screen*, identif*, assess*, predict*  **NEAR/3 or N3**  Need(s), tool*, instrument(s), evaluat*, questionnaire*, survey*, check list*, checklist*, score(s), scoring, scale*, detect*, decision tool*, decision aid*, decision tree*, decision support*, algorithm*, indicator*, criteria, criterion, guidelines(s), guide(s), policy, policies, framework*, pathway* |

**Bibliographic Database Search Strategies**

**Search date:** 26^th^ February 2024

**Table 2. MEDLINE search strategy (EBSCO host)**

| **Search** | **Query** | **Records retrieved** |
| --- | --- | --- |
| **#1** | **MeSH Terms:** "Hospice and Palliative Care Nursing" OR "Palliative Medicine" OR "Palliative Care" OR "Terminal Care+"  **OR**  **Title/Abstract:** palliat* OR "end of life" OR "end-of-life" OR "EOL" OR terminal OR terminally OR hospic* OR dying OR "last year of life" | [**681,555**](https://pubmed.ncbi.nlm.nih.gov/?term=%28palliat%2A%5BTitle%2FAbstract%5D+OR+%22end+of+life%22%5BTitle%2FAbstract%5D+OR+end-of-life%5BTitle%2FAbstract%5D+OR+EOL%5BTitle%2FAbstract%5D+OR+terminal%2A%5BTitle%2FAbstract%5D+OR+hospic%2A%5BTitle%2FAbstract%5D+OR+dying%5BTitle%2FAbstract%5D+OR+death%5BTitle%2FAbstract%5D+OR+%22last+year+of+life%22%5BTitle%2FAbstract%5D%29+OR+%28%22Palliative+Medicine%22%5BMesh%5D+OR+%22Palliative+Care%22%5BMesh%5D+OR+%22Terminal+Care%22%5BMesh%5D%29&sort=relevance) |
| **#2** | **MeSH Terms:** "Nursing Homes+" OR "Residential Facilities+" OR "Assisted Living Facilities" OR "Homes for the Aged"  **OR**  **Title/Abstract:** "nursing home*" OR "long term care" OR "long-term care" OR "retirement home*" OR "residential care" OR "residential home" OR "residential homes" OR "residential facility" OR "residential facilities" OR "assisted living" OR "assisted-living" OR "old-age home" OR "old-age homes" OR "homes for the aged" OR "care home" OR "care homes" | [**94,754**](https://pubmed.ncbi.nlm.nih.gov/?term=%28%28%28%28%22Nursing+Homes%22%5BMesh%5D%29+OR+%22Residential+Facilities%22%5BMesh%5D%29+OR+%22Assisted+Living+Facilities%22%5BMesh%5D%29+OR+%22Homes+for+the+Aged%22%5BMesh%5D%29+OR+%28%22Nursing+home%2A%22%5BTitle%2FAbstract%5D+OR+%22long+term+care%22%5BTitle%2FAbstract%5D+OR+%22long-term+care%22%5BTitle%2FAbstract%5D+OR+%22retirement+home%2A%22%5BTitle%2FAbstract%5D+OR+%22residential+care%22%5BTitle%2FAbstract%5D+OR+%22residential+home%2A%22%5BTitle%2FAbstract%5D+OR+%22residential+facilit%2A%22%5BTitle%2FAbstract%5D+OR+%22assisted+living%22%5BTitle%2FAbstract%5D+OR+assisted-living%5BTitle%2FAbstract%5D+OR+%22old-age+home%22%5BTitle%2FAbstract%5D+OR+%22homes+for+the+aged%22%5BTitle%2FAbstract%5D%29&sort=relevance) |
| **#3** | **MeSH Terms:** "Needs Assessment+" OR "Nursing Assessment+" OR "Practice Guideline"  **OR**  **Title/Abstract:** (screen* OR identif* OR assess* OR predict*) N3 (need OR needs OR tool* OR instrument OR instruments OR evaluat* OR questionnaire* OR survey* OR "check list* OR checklist* OR score OR scores OR scoring OR scale* OR detect* OR "decision tool*" OR "decision aid*"OR "decision tree*" OR "decision support*" OR algorithm* OR indicator* OR criteria OR criterion OR guideline OR guidelines OR guide OR guides OR policy OR policies OR framework* OR pathway* | [**1,198,651**](https://pubmed.ncbi.nlm.nih.gov/?term=%28%22Needs+Assessment%22%5BMesh%5D+OR+%22Geriatric+Assessment%22%5BMesh%5D+OR+%22Nursing+Assessment%22%5BMesh%5D%29+OR+%28%28screen%2A%5BTitle%2FAbstract%5D+OR+identif%2A%5BTitle%2FAbstract%5D+OR+assess%2A%29+N3+%28need%2A%5BTitle%2FAbstract%5D+OR+tool%2A%5BTitle%2FAbstract%5D+OR+instrument%2A%5BTitle%2FAbstract%5D+OR+evaluat%2A%5BTitle%2FAbstract%5D+OR+questionnaire%2A%5BTitle%2FAbstract%5D+OR+survey%2A%5BTitle%2FAbstract%5D+OR+measur%2A%5BTitle%2FAbstract%5D+OR+%22check+list%22%5BTitle%2FAbstract%5D+OR+checklist%5BTitle%2FAbstract%5D+OR+scor%2A%5BTitle%2FAbstract%5D+OR+scale%2A%5BTitle%2FAbstract%5D+OR+trigger%2A%5BTitle%2FAbstract%5D+OR+refer%2A%5BTitle%2FAbstract%5D+OR+consult%2A%5BTitle%2FAbstract%5D+OR+detect%2A%5BTitle%2FAbstract%5D+OR+%22decision+tool%2A%22%5BTitle%2FAbstract%5D+OR+%22decision+aid%2A%22%5BTitle%2FAbstract%5D+OR+%22decision+tree%2A%22%5BTitle%2FAbstract%5D+OR+%22decision+support%2A%22%5BTitle%2FAbstract%5D+OR+algorithm%2A%5BTitle%2FAbstract%5D%29%29&sort=relevance) |
| **#4** | #1 AND #2 AND #3 | **749** |

**Table 3. Embase search strategy**

| **Search** | **Query** | **Records retrieved** |
| --- | --- | --- |
| **#1** | **Emtree Subject Headings:** "palliative therapy"/exp OR "palliative nursing"/exp  **OR**  **Title/Abstract:** palliat* OR "end of life" OR "end-of-life" OR "EOL" OR terminal OR terminally OR hospic* OR dying OR "last year of life" | [**809,703**](https://pubmed.ncbi.nlm.nih.gov/?term=%28palliat%2A%5BTitle%2FAbstract%5D+OR+%22end+of+life%22%5BTitle%2FAbstract%5D+OR+end-of-life%5BTitle%2FAbstract%5D+OR+EOL%5BTitle%2FAbstract%5D+OR+terminal%2A%5BTitle%2FAbstract%5D+OR+hospic%2A%5BTitle%2FAbstract%5D+OR+dying%5BTitle%2FAbstract%5D+OR+death%5BTitle%2FAbstract%5D+OR+%22last+year+of+life%22%5BTitle%2FAbstract%5D%29+OR+%28%22Palliative+Medicine%22%5BMesh%5D+OR+%22Palliative+Care%22%5BMesh%5D+OR+%22Terminal+Care%22%5BMesh%5D%29&sort=relevance) |
| **#2** | **Emtree Subject Headings:** "nursing home"/exp  **OR**  **Title/Abstract:** "nursing home*" OR "long term care" OR "long-term care" OR "retirement home*" OR "residential care" OR "residential home" OR "residential homes" OR "residential facility" OR "residential facilities" OR "assisted living" OR "assisted-living" OR "old-age home" OR "old-age homes" OR "homes for the aged" OR "care home" OR "care homes" | **74,963** |
| **#3** | **Emtree Subject Headings:** screening/exp OR "practice guideline"/exp OR "clinical pathway"/exp OR "health care policy"  **OR**  **Title/Abstract:** (screen* OR identif* OR assess* OR predict*) NEAR/3 (need OR needs OR tool* OR instrument OR instruments OR evaluat* OR questionnaire* OR survey* OR "check list* OR checklist* OR score OR scores OR scoring" OR scale* OR detect* OR "decision tool*" OR "decision aid*"OR "decision tree*" OR "decision support*" OR algorithm* OR indicator* OR criteria OR criterion OR guideline OR guidelines OR guide OR guides OR policy OR policies OR framework* OR pathway* | **2,749,747** |
| **#4** | #1 AND #2 AND #3 | **1059** |

**Table 4. CINAHL search strategy (EBSCO host)**

| **Search** | **Query** | **Records retrieved** |
| --- | --- | --- |
| **#1** | **CINAHL Subject Headings:** "Palliative Care Nursing" OR "Palliative Medicine" OR "Palliative Care"  **OR**  **Title/Abstract:** palliat* OR "end of life" OR "end-of-life" OR "EOL" OR terminal OR terminally OR hospic* OR dying OR "last year of life" | [**43,310**](https://pubmed.ncbi.nlm.nih.gov/?term=%28palliat%2A%5BTitle%2FAbstract%5D+OR+%22end+of+life%22%5BTitle%2FAbstract%5D+OR+end-of-life%5BTitle%2FAbstract%5D+OR+EOL%5BTitle%2FAbstract%5D+OR+terminal%2A%5BTitle%2FAbstract%5D+OR+hospic%2A%5BTitle%2FAbstract%5D+OR+dying%5BTitle%2FAbstract%5D+OR+death%5BTitle%2FAbstract%5D+OR+%22last+year+of+life%22%5BTitle%2FAbstract%5D%29+OR+%28%22Palliative+Medicine%22%5BMesh%5D+OR+%22Palliative+Care%22%5BMesh%5D+OR+%22Terminal+Care%22%5BMesh%5D%29&sort=relevance) |
| **#2** | **CINAHL Subject Headings:** "Nursing Homes+" OR "Residential Facilities+"  **OR**  **Title/Abstract:** "nursing home*" OR "long term care" OR "long-term care" OR "retirement home*" OR "residential care" OR "residential home" OR "residential homes" OR "residential facility" OR "residential facilities" OR "assisted living" OR "assisted-living" OR "old-age home" OR "old-age homes" OR "homes for the aged" OR "care home" OR "care homes" | [**68,613**](https://pubmed.ncbi.nlm.nih.gov/?term=%28%28%28%28%22Nursing+Homes%22%5BMesh%5D%29+OR+%22Residential+Facilities%22%5BMesh%5D%29+OR+%22Assisted+Living+Facilities%22%5BMesh%5D%29+OR+%22Homes+for+the+Aged%22%5BMesh%5D%29+OR+%28%22Nursing+home%2A%22%5BTitle%2FAbstract%5D+OR+%22long+term+care%22%5BTitle%2FAbstract%5D+OR+%22long-term+care%22%5BTitle%2FAbstract%5D+OR+%22retirement+home%2A%22%5BTitle%2FAbstract%5D+OR+%22residential+care%22%5BTitle%2FAbstract%5D+OR+%22residential+home%2A%22%5BTitle%2FAbstract%5D+OR+%22residential+facilit%2A%22%5BTitle%2FAbstract%5D+OR+%22assisted+living%22%5BTitle%2FAbstract%5D+OR+assisted-living%5BTitle%2FAbstract%5D+OR+%22old-age+home%22%5BTitle%2FAbstract%5D+OR+%22homes+for+the+aged%22%5BTitle%2FAbstract%5D%29&sort=relevance) |
| **#3** | **CINAHL Subject Headings:** "Clinical Assessment Tools+" OR "Needs Assessment" OR "Practice Guidelines" OR "Health Policy+"  **OR**  **Title/Abstract:** (screen* OR identif* OR assess* OR predict*) N3 (need OR needs OR tool* OR instrument OR instruments OR evaluat* OR questionnaire* OR survey* OR "check list* OR checklist* OR score OR scores OR scoring OR scale* OR detect* OR "decision tool*" OR "decision aid*"OR "decision tree*" OR "decision support*" OR algorithm* OR indicator* OR criteria OR criterion OR guideline OR guidelines OR guide OR guides OR policy OR policies OR framework* OR pathway* | [**757,596**](https://pubmed.ncbi.nlm.nih.gov/?term=%28%22Needs+Assessment%22%5BMesh%5D+OR+%22Geriatric+Assessment%22%5BMesh%5D+OR+%22Nursing+Assessment%22%5BMesh%5D%29+OR+%28%28screen%2A%5BTitle%2FAbstract%5D+OR+identif%2A%5BTitle%2FAbstract%5D+OR+assess%2A%29+N3+%28need%2A%5BTitle%2FAbstract%5D+OR+tool%2A%5BTitle%2FAbstract%5D+OR+instrument%2A%5BTitle%2FAbstract%5D+OR+evaluat%2A%5BTitle%2FAbstract%5D+OR+questionnaire%2A%5BTitle%2FAbstract%5D+OR+survey%2A%5BTitle%2FAbstract%5D+OR+measur%2A%5BTitle%2FAbstract%5D+OR+%22check+list%22%5BTitle%2FAbstract%5D+OR+checklist%5BTitle%2FAbstract%5D+OR+scor%2A%5BTitle%2FAbstract%5D+OR+scale%2A%5BTitle%2FAbstract%5D+OR+trigger%2A%5BTitle%2FAbstract%5D+OR+refer%2A%5BTitle%2FAbstract%5D+OR+consult%2A%5BTitle%2FAbstract%5D+OR+detect%2A%5BTitle%2FAbstract%5D+OR+%22decision+tool%2A%22%5BTitle%2FAbstract%5D+OR+%22decision+aid%2A%22%5BTitle%2FAbstract%5D+OR+%22decision+tree%2A%22%5BTitle%2FAbstract%5D+OR+%22decision+support%2A%22%5BTitle%2FAbstract%5D+OR+algorithm%2A%5BTitle%2FAbstract%5D%29%29&sort=relevance) |
| **#4** | #1 AND #2 AND #3 | **788** |

**Table 5. Web of Science search strategy**

| **Search** | **Query** | **Records retrieved** |
| --- | --- | --- |
| **#1** | **Title/Abstract:** palliat* OR "end of life" OR "end-of-life" OR "EOL" OR terminal OR terminally OR hospic* OR dying OR "last year of life" | [**1,455,364**](https://pubmed.ncbi.nlm.nih.gov/?term=%28palliat%2A%5BTitle%2FAbstract%5D+OR+%22end+of+life%22%5BTitle%2FAbstract%5D+OR+end-of-life%5BTitle%2FAbstract%5D+OR+EOL%5BTitle%2FAbstract%5D+OR+terminal%2A%5BTitle%2FAbstract%5D+OR+hospic%2A%5BTitle%2FAbstract%5D+OR+dying%5BTitle%2FAbstract%5D+OR+death%5BTitle%2FAbstract%5D+OR+%22last+year+of+life%22%5BTitle%2FAbstract%5D%29+OR+%28%22Palliative+Medicine%22%5BMesh%5D+OR+%22Palliative+Care%22%5BMesh%5D+OR+%22Terminal+Care%22%5BMesh%5D%29&sort=relevance) |
| **#2** | **Title/Abstract:** "nursing home*" OR "long term care" OR "long-term care" OR "retirement home*" OR "residential care" OR "residential home" OR "residential homes" OR "residential facility" OR "residential facilities" OR "assisted living" OR "assisted-living" OR "old-age home" OR "old-age homes" OR "homes for the aged" OR "care home" OR "care homes" | **72,118** |
| **#3** | **Title/Abstract:** (screen* OR identif* OR assess* OR predict*) NEAR/3 (need OR needs OR tool* OR instrument OR instruments OR evaluat* OR questionnaire* OR survey* OR "check list* OR checklist* OR score OR scores OR scoring" OR scale* OR detect* OR "decision tool*" OR "decision aid*"OR "decision tree*" OR "decision support*" OR algorithm* OR indicator* OR criteria OR criterion OR guideline OR guidelines OR guide OR guides OR policy OR policies OR framework* OR pathway* | [**1,567,**](https://pubmed.ncbi.nlm.nih.gov/?term=%28%22Needs+Assessment%22%5BMesh%5D+OR+%22Geriatric+Assessment%22%5BMesh%5D+OR+%22Nursing+Assessment%22%5BMesh%5D%29+OR+%28%28screen%2A%5BTitle%2FAbstract%5D+OR+identif%2A%5BTitle%2FAbstract%5D+OR+assess%2A%29+N3+%28need%2A%5BTitle%2FAbstract%5D+OR+tool%2A%5BTitle%2FAbstract%5D+OR+instrument%2A%5BTitle%2FAbstract%5D+OR+evaluat%2A%5BTitle%2FAbstract%5D+OR+questionnaire%2A%5BTitle%2FAbstract%5D+OR+survey%2A%5BTitle%2FAbstract%5D+OR+measur%2A%5BTitle%2FAbstract%5D+OR+%22check+list%22%5BTitle%2FAbstract%5D+OR+checklist%5BTitle%2FAbstract%5D+OR+scor%2A%5BTitle%2FAbstract%5D+OR+scale%2A%5BTitle%2FAbstract%5D+OR+trigger%2A%5BTitle%2FAbstract%5D+OR+refer%2A%5BTitle%2FAbstract%5D+OR+consult%2A%5BTitle%2FAbstract%5D+OR+detect%2A%5BTitle%2FAbstract%5D+OR+%22decision+tool%2A%22%5BTitle%2FAbstract%5D+OR+%22decision+aid%2A%22%5BTitle%2FAbstract%5D+OR+%22decision+tree%2A%22%5BTitle%2FAbstract%5D+OR+%22decision+support%2A%22%5BTitle%2FAbstract%5D+OR+algorithm%2A%5BTitle%2FAbstract%5D%29%29&sort=relevance)**406** |
| **#4** | #1 AND #2 AND #3 | **602** |

**Table 6. PsycINFO search strategy (EBSCO host)**

| **Search** | **Query** | **Records retrieved** |
| --- | --- | --- |
| **#1** | **APA Subject Headings:** "Palliative Care"  **OR**  **Title/Abstract:** palliat* OR "end of life" OR "end-of-life" OR "EOL" OR terminal OR terminally OR hospic* OR dying OR "last year of life" | **54,828** |
| **#2** | **APA Subject Headings:** "Nursing Home Residents" OR "Nursing Homes"  **OR**  **Title/Abstract:** "nursing home*" OR "long term care" OR "long-term care" OR "retirement home*" OR "residential care" OR "residential home" OR "residential homes" OR "residential facility" OR "residential facilities" OR "assisted living" OR "assisted-living" OR "old-age home" OR "old-age homes" OR "homes for the aged" OR "care home" OR "care homes" | **27,972** |
| **#3** | **APA Subject Headings:** Screening OR "Screening Tests"  **OR**  **Title/Abstract:** (screen* OR identif* OR assess* OR predict*) N3 (need OR needs OR tool* OR instrument OR instruments OR evaluat* OR questionnaire* OR survey* OR "check list* OR checklist* OR score OR scores OR scoring" OR scale* OR detect* OR "decision tool*" OR "decision aid*"OR "decision tree*" OR "decision support*" OR algorithm* OR indicator* OR criteria OR criterion OR guideline OR guidelines OR guide OR guides OR policy OR policies OR framework* OR pathway* | **296,724** |
| **#4** | #1 AND #2 AND #3 | **195** |

**Table 7. APA PsycArticles search strategy (EBSCO host)**

| **Search** | **Query** | **Records retrieved** |
| --- | --- | --- |
| **#1** | **APA Subject Headings:** "Palliative Care"  **OR**  **Title/Abstract:** palliat* OR "end of life" OR "end-of-life" OR "EOL" OR terminal OR terminally OR hospic* OR dying OR "last year of life" | **846** |
| **#2** | **APA Subject Headings:** "Nursing Home Residents" OR "Nursing Homes"  **OR**  **Title/Abstract:** "nursing home*" OR "long term care" OR "long-term care" OR "retirement home*" OR "residential care" OR "residential home" OR "residential homes" OR "residential facility" OR "residential facilities" OR "assisted living" OR "assisted-living" OR "old-age home" OR "old-age homes" OR "homes for the aged" OR "care home" OR "care homes" | **355** |
| **#3** | **APA Subject Headings:** Screening OR "Screening Tests"  **OR**  **Title/Abstract:** (screen* OR identif* OR assess* OR predict*) N3 (need OR needs OR tool* OR instrument OR instruments OR evaluat* OR questionnaire* OR survey* OR "check list* OR checklist* OR score OR scores OR scoring" OR scale* OR detect* OR "decision tool*" OR "decision aid*"OR "decision tree*" OR "decision support*" OR algorithm* OR indicator* OR criteria OR criterion OR guideline OR guidelines OR guide OR guides OR policy OR policies OR framework* OR pathway* | **10,560** |
| **#4** | #1 AND #2 AND #3 | **1** |

**Grey Literature Databases Search Strategies**

**Database/website:** Clinicaltrials.gov

**URL:** https://www.clinicaltrials.gov/

**Search date:** 7^th^ March 2024

**Search strategy:**

(palliative OR "end of life" OR end-of-life OR EOL OR terminal OR terminally OR hospice OR dying OR "last year of life") AND ("Nursing home" OR "nursing homes" OR "long term care" OR "long-term care" OR "retirement home" OR "residential care" OR "residential home" OR "residential homes" OR "residential facility" "residential facilities" OR "assisted living" OR assisted-living OR "old-age home" OR "old-age homes" OR "homes for the aged" OR "care home" OR "care homes") AND (screen OR identify OR assess OR predict OR guideline OR guidelines OR guide OR guides OR policy OR policies OR framework OR pathway)

**Total records:** 2633 records

**Included for full text review:** 3 records

**Database/website:** CareSearch

**URL:** https://www.caresearch.com.au/

**Search date:** 4^th^ March 2024

**Search strategy:**

(palliative OR "end of life" OR end-of-life OR EOL OR terminal OR terminally OR hospice OR dying OR "last year of life") AND ("Nursing home" OR "nursing homes" OR "long term care" OR "long-term care" OR "retirement home" OR "residential care" OR "residential home" OR "residential homes" OR "residential facility" "residential facilities" OR "assisted living" OR assisted-living OR "old-age home" OR "old-age homes" OR "homes for the aged" OR "care home" OR "care homes") AND (screen OR identify OR assess OR predict OR guideline OR guidelines OR guide OR guides OR policy OR policies OR framework OR pathway)

**Total records:** 4231 records

**Included for full text review:** 3 records

**Database/website:** Trip

**URL:** https://www.tripdatabase.com/

**Search date:** 25^th^ March 2024

**Search strategy:** Palliative AND “nursing home” AND screening

**Total records:** 634 records

**Included for full text review:** 1 record

**Database/website:** GuidelineCentral

**URL:** https://www.guidelinecentral.com/guidelines/

**Search date:** 22^nd^ March 2024

**Search strategy:**

palliative nursing home = 14 records

palliative long-term care = 15 records

palliative care home = 21 records

end of life nursing home = 24 records

end of life care home = 22 records

end of life long-term care = 22 records

**Total records:** 118 records (multiple duplicates)

**Included for full text review:** 0 records

**Database/website:** Guidelines International Network

**URL:** https://www.guidelinecentral.com/guidelines/

**Search date:** 25th March 2024

**Search strategy:**

palliative nursing home = 1 record

palliative long-term care = 1 record

palliative care home = 2 records

end of life nursing home = 0 records

end of life care home = 0 records

end of life long-term care = 0 records

palliative = 89 records

end of life = 12 records

**Total records:** 105 records (multiple duplicates)

**Included for full text review:** 0 records

**Database/website:** NICE Website

**URL:** https://www.nice.org.uk/

**Search date:** 4^th^ March 2024

**Search strategy:**

Palliative and “nursing home” = 30 records

Palliative and “long-term care” = 95 records

palliative and “care home” = 68 records

“end of life” and “nursing home” = 69 records

“end of life” and “care home” = 214 records

“end of life” and “long-term care” = 14 records

**Total records:** 476 (multiple duplicates)

**Included for full text review:** 1 record
