# Supplementary material for: Identifying unmet palliative care needs of nursing home residents: A scoping review
Source: PLoS One. 2025 Feb 25;20(2):e0319403. doi: 10.1371/journal.pone.0319403 (PMC11856323; doi:10.1371/journal.pone.0319403)
Supplement: S1 Table — (DOCX) [file pone.0319403.s003.docx]

**Data extraction table 1a: Methods of identifying unmet palliative care needs**

| **Study Details** | | **Methods Characteristics** | | **Assessment** | |
| --- | --- | --- | --- | --- | --- |
| **a) Reference**  **b) Country**  **c) Time frame**  **d) Design** | **a) Study aim**  **b) Sample**  **c) Resident demographics** | **a) Method/format**  **b) Purpose**  **c) Specificity to disease** | **Indicators** | **a) Assessor**  **b) Recommended frequency of assessment**  **c) Time needed to complete** | **a) Scoring system**  **b) Referral pathway** |
| **a)** Aranha et al.  (2020)  **b)** USA  **c)** Last year of life (predicting)  **d)** Quantitative retrospective and prospective study | **a)** To assess frailty and palliative performance scores in long-term care residents and to establish whether the Clinical Frailty Scale (CFS) and Palliative Performance Scale (PPS) can predict re hospitalisations  **b)** 171 residents from 2 long term care facilities  **c)** Mean age of 73.1 years  Female (52.6%) | **a)** CFS and PPS  **b) CFS:**  To assess an individual’s level of frailty  **PPS:**  To assess an individual’s functional ability and end of life development  **c)** Non disease specific | **CFS:**   - Lessened activity - Reduced mobility - Weight loss - Reduced handgrip Strength - Fatigue   **PPS:**   - Walking ability - Activity - Degree of disease progression - Oral consumption - Level of consciousness   **Hospital readmissions:**  CFS (p = .002)  PPS (p = .001) | **a)** Research assistant  **b)** Not reported  **c)** Not reported | **a) CFS:**  Patient scores from very fit (1) to severely frail (7)  **PPS:**  Healthy with full ability to walk = 100%  Dead = 0%  **b)** Not reported |
| **a)** Badger et al.  (2009)  **b)** England  **c)** Not specified  **d)** Quantitative pre-post survey design | **a)** “The aim of the study was to evaluate the impact of the introduction of the GSF into care homes” p. 503  **b)** 95 NH (only 49 completed pre and post survey)  **c)** Not reported | **a)** Gold Standards Framework in Care Homes  **b)** To assist care home staff to identify residents who may benefit from palliative or end of life care and to complete a holistic assessment of the person  **c)** Non disease specific | Not reported  “At follow-up, there were significant changes in the proportions of homes that had **systems for identifying residents** in need of end-of-life care, had care coordinators and were routinely undertaking advanced care planning” p. 508 | **a)** NH staff  **b)** Not reported  **c)** Intervention introduced over a period of 8 months | **a)** Not reported  **b)** ACP, DNR, needs assessment and management |
| **a)** Bußmann and Pomorin  (2023)  **b)** Germany  **c)** “Last phase of life” (staff identification)  **d)** Qualitative design using semi structured interviews | **a)** To investigate palliative care related issues in daily practice and to identify areas for improving palliative care for nursing home residents  **b)** 14 staff from 2 nursing homes  **c)** N/A | **a)** Staff assessment/ recognition  **b)** To identify palliative care needs  **c)** Non disease specific | - Residents’ diagnosis - Staff perception of resident based on assessments - Residents wishes - Residents’ opinion   As well as:   - Changed breathing - Withdrawing socially - Loss of appetite | **a)** Nurses, carers, and managers  **b)** Not reported  **c)** N/A | **a)** N/A  **b)**  **1.** Potential palliative care need identified  **2.** Nursing specialist assesses  **3.** No need identified:  continue to monitor  **4.** Need identified:  Alleviate symptoms  GP consult  Update care plan  Query if need for external PC support  Family meeting  Educate resident and family |
| **a)** Casarett et al.  (2005)  **b)** USA  **c)** Last 6 months of life (predicting)  **d)** Quantitative RCT | **a)** To evaluate whether a communication intervention increases hospice referrals and improve end-of-life care for residents.  **b)** 205 residents from 3 nursing homes  **c)** Mean age of 84 years  Female (75%)  Dementia (62%)  CHF (22%)  Chronic Lung Disease (17%) | **a)** Interview (resident and/or surrogate) and referral to GP if appropriate  **b)** To increase the number of hospice referrals and to enhance the end-of-life care provided in care homes  **c)** Non disease specific | **Suitability for hospice:**   - Person or surrogate stated the want for comfort care - A DNR and ACD indicating refusal of mechanical ventilation   **Palliative care needs (based on GDI of MSAS):**   - Pain - Constipation - Loss of appetite - Low energy - Drowsiness - Xerostomia - Sadness - Worry - Nervousness - Anxiety   **Other needs identified:**  Required more nursing care, required help with personal care, lack of advance care planning, counselling and psychological care, spiritual care | **a)** Researcher (but could be given by any member of the healthcare team)  **b)** Not reported  **c)** 5 to 10 minutes | **a)** Person must fulfil hospice suitability criteria plus 1 palliative care need to be referred to GP  Referred to hospice if GP determined the resident to have a prognosis of less than 6 months  **b)** Hospice referral, residents got the option for palliative care or for life-preserving treatment |
| **a)** Chapman et al.  (2016)  **b)** Australia  **c)** Last 6 months of life (staff identification)  **d)** Quantitative quasi-experimental design | **a)** To evaluate if implementing a model of specialist palliative care decreased acute care admissions and hospital deaths  **b)** 104 residents (intervention) from 4 RACF and 173 historical controls  **c)** Intervention:  Mean age of 89 years  Females (75%)  Dementia (40%)  CHF (14%)  CVA (14%)  Control:  Mean age of 86 years  Females (62%)  Dementia (29%) CHF (12%)  Frailty (15%) | **a)** Palliative Care Needs Rounds  **b)** To enhance communication between the SPC and the RACF teams and to facilitate care planning and assessing residents suitable for PC  **c)** Non disease specific | - Surprise question (6 months) - Mean age-modified Charlson score of 6.4 | **a)** RACF staff and Palliative Care Nurse Practitioner  **b)** Monthly  **c)** Not reported | **a)** Not reported  **b)** Advance care planning, symptom management, GP referral, specialist palliative care referral, case conferences, anticipatory prescribing |
| **a)** Comart et al.  (2012)  **b)** USA  **c)** Last 1-2 years of life (actual)  **d)** Quantitative quasi-experimental design | **a)** To evaluate if implementing a palliative care consultation service would improve treatment and outcomes for residents in a long term care facility.  **b)** 150 intervention residents (IR) and  150 historical controls (HC)  **c) Females:**  IR (71.2%)  HC (74.4%)  **Age at death:**  Mean age of 89.1 years (IR)  Mean age of 89.9 years (HC) | **a)** Staff education and training and PC consultations  **b)** To improve residents’ outcomes such as ED admissions and symptom management  **c)** Non disease specific | Eligibility criteria not reported.  **Minimum Data Set:**   - ADL score: 4.3 (IR), 4.5 (HC) - CPS score: 3.4 (IR), 3.5 (HC) - Depression rating scale: 2.8 (IR), 2.5 (HC) - Pain Index Scale: 0.98 (IR), 0.98 (HC) - Delirium: 65.6% (IR), 73.6% (HC)   **Other needs:**  Falls, pressure ulcers, dyspnoea, weight loss, quantity of medications, and hospital admissions. | **a)** LTC staff and PC team (PC doctor, CNS, chaplain, social worker, and a psychologist)  **b)** Bi-weekly meetings  **c)** Not reported | **a)** Not reported  **b)** Symptom assessment and management, help with deciding about care, full needs assessment including spiritual/religious needs, goals and preferences of care, education of family/resident, help with developing coping mechanisms |
| **a)** Davis et al.  (2023)  **b)** Australia  **c)** Last year of life (predicted)  **d)** Qualitative design using interviews and focus groups | **a)** To determine the feasibility of the model and factors influencing its successful implementation in aged care homes.  **b)** 14 care workers, 13 HCP, 10 managers  **c)** N/A | **a)** Palliative care needs assessment and referral procedure  **b)** To enhance PC for residents who are nearing end of life as well as supporting their relatives, and care staff  **c)** Non disease specific | - Australian Modified Karnofksy Performance Scale (AKPS) of 40 or below - Supportive and Palliative Care Indicators Tool (SPICT) - Surprise question (12 months)+ | **a)** Care home staff (nurses, healthcare assistants)  **b)** Every 3 months or if indicated  **c)** Not reported | **a)** Must have all 3 indicators to move to full assessment  **b)** Family meeting to discuss care goals, palliative assessment using 5 PCOC tools (AKPS, PCOC SAS, PCPSS, Palliative Care Phase, RUG-ADL), ACP/DNR, medication review |
| **a)** Estabrooks et al.  (2015)  **b)** Canada  **c)** Last year of life (actual)  **d)** Quantitative retrospective analysis of longitudinal survey data | **a)** To investigate how the organizational context of a nursing home influences symptom burden and to make comparisons of symptom burden between residents with and without dementia in their final year of life.  **b)** 2635 residents with dementia and 1012 residents without dementia from 36 nursing homes  **c)** Female (65.8%)  Dementia (72.3%)  Stroke (22.1%)  HF (17.8%)  **Age at death:** mean of 88 years  **Length of residence before death:** mean of 3.5 years | **a)** Resident Assessment Instrument- Minimum Data Set (RAI-MDS)  **b)** Identification of symptoms of residents in their last year of life  **c)** Dementia and nondementia | **Three months before death:**   - Challenging behaviour:   Dementia (most common symptom for this group) (40.2%)  Non-dementia (20.6%)   - Delirium:   Non-dementia (most common symptom for this group) (31%) Dementia (29.2%)   - UTI (least common symptom for both groups):   Dementia (9.1%)  Non-dementia (10%)   - Increase in pressure ulcers:   Dementia (4.6% to 9.5%)  Non-dementia (5.3% to 10.7%)  **Dementia:**   - More challenging behaviour - More antipsychotics   **Non-dementia:**   - More difficulty breathing - More pain   **Both groups:**   - ADL score of 15+ (82%) - CPS score of 4+ (55.9%) - Displaying any aggressive behaviour (53%) - CHESS 2+ (49%) - Depression Rating Scale of 3+ (37.8%) | **a)** NH staff  **b)** RAI-MDS collected every 3 months  **c)** Not reported | **a)** N/A  **b)** Not reported |
| **a)** Esteban-Burgos et al.,  (2021)  **b)** Spain  **c)** “Limited life expectancy” (predicted)  **d)** Quantitative observational, descriptive, cross-sectional study | **a)** To assess resident’s palliative needs and to identify new indicators of advanced chronic illnesses  **b)** 149 residents from 7 nursing homes  **c)** Mean age of 84.47 years  Female (67.1%)  Dementia (45.6%)  CHD (38.3%)  Chronic Pulmonary Disease (22.8%) | **a)** Necesidades Paliativas  (NECPAL) Version 3.1 (screening tool)  **b)** The purpose of the tool is early identification of people with palliative care needs  **c)** Disease specific indicators for: cancer, chronic lung disease, CHD, chronic liver disease, chronic renal disease, CVA, dementia, frailty, neurodegenerative diseases (MND, MS, ALS Parkinsons), AIDS | - Surprise question (12 months) [*authors recommend replacing with the Frail VIG Index] - Resident states need for PC - HCP states need for PC - Nutritional deterioration - Functional deterioration - Cognitive deterioration - Severe dependence - “geriatric syndromes” - Consistent symptoms - Distress +/- “Severe Adaptive Disorder” - “Severe Social Vulnerability” - Multiple comorbidities - “Use of resources” | **a)** Healthcare professionals  **b)** Not reported  **c)** Not reported | **a)** **1.** Surprise question (12 months) negative = NECPAL negative  **2.** Surprise question (12 months) positive = further evaluation, resident must have at least 1 additional parameter  **b)** Not reported (however tool does have its own pathway) |
| **a)** Forbat et al.  (2018)  **b)** Australia  **c)** “Final months of life” (predicted)  **d)** Qualitative grounded theory ethnographic study | **a)** To develop a checklist to support the integration of palliative care by specialists in nursing homes  **b)** 97 residents from 4 residential facilities  **c)** N/A | **a)** Palliative Care Needs Rounds Checklist (screening tool)  **b)** To identify residents with potential palliative care needs and prompt further assessments or review of care plan  **c)** Non disease specific | - Surprise question (6 months) - Physical or cognitive deterioration or worsening of symptoms in the previous month - No care plan for the last 6 months of life or no advance care plan - Disagreements with the residents’ relatives about treatment and plan of care - Resident was transferred to the facility for end of life care | **a)** RN, manager, team leader, advance nurse practitioner  **b)** Monthly  **c)** Hour long meetings | **a)** Resident must trigger at least 1 of 5 triggers to be discussed at the needs round  **b)** **1****.** Identify diagnosis and comorbidities  **2.** Identify palliative care needs  **3.** Identify staff concerns  **4.** Identify residents support structure  **5.** Educate staff on potential scenarios specific to that resident (e.g. recognising deterioration and dying)  **6.** Review medications  **7.** Ensure resident has a proxy decision maker if necessary  **8.** Consider ACP  **9****.** Consider planning a case conference  **10.** Consider outside referrals (e.g. pastoral care, wound care)  **11.** Consider referral to specialist palliative care |
| **a)** Forbat et al.  (2020)  **b)** Australia  **c)** “Last months of life” (predicted)  **d)** Quantitative prospective step wedged RCT | **a)** To evaluate if Palliative Care Needs Rounds reduce the duration of stay for residents when hospitalised  **b)** 567 out of 1700 residents from 12 RACF  **c)** Mean age of 85 years  Female (64%)  Dementia/Parkinson (34%)  CVD (13%)  Frailty (7.5%) | **a)** Palliative Care Needs Rounds Checklist (screening tool)  **b)** To identify residents who may die, have the greatest symptom encumbrance, and have no advance care planning  **c)** Non disease specific | - Surprise question (6 months) - Physical/ cognitive deterioration in previous month - Lack of ACP or plan for last 6 months before death - Disagreements amongst the resident relatives around treatment/care - A direct transfer for end of life care | **a)** Specialist PC staff (nurse practitioners and clinical nurse consultant) and care home staff (nurses, HCAs, managers, activities coordinators)  **b)** Once a month  **c)** 1 hour meetings | **a)** Resident must have 1+ indicators to be discussed  **b)** ACP, anticipatory prescribing, case conferences, identifying healthcare proxy |
| **a)** Giuffrida  (2015)  **b)** USA  **c)** Not specified  **d)** Description of the development of a palliative care program | **a)** To improve the number of residents receiving palliative care, the number of advance care directives, and palliative care referrals from the hospital and to decrease hospital re admissions  **b)** Not reported  **c)** Not reported | **a)** Establishing a palliative care committee, staff education, introducing a list of residents receiving PC, and discussing deteriorating residents every morning  **b)** To improve the number of residents in palliative care, improve amount of documented ACDs, lower the number of repeated hospitalisations, and improve the amount of PC referrals  **c)** Non disease specific | - Weight loss - Pneumonia - Recurrent hospital admissions | **a)** Nurses, doctors, dietitians, activity coordinator, pastoral care, volunteers  **b)** Deteriorating residents discussed daily  **c)** Not reported | **a)** Not reported  **b)** Advance Care Directive, DNR, allocation of a healthcare proxy, Do Not Hospitalise Order, needs assessment, hospice referral |
| **a)** Grbich et al.  (2005)  **b)** Australia  **c)** Last 6 months of life (predicted)  **d)** Mixed method design | **a)** To establish eligibility criteria for palliative care for residents without cancer  **b)** Stage 1: 298 RACF  Stage 2: 69 residents (16 of those died)  **c)** 16 that died:  Haematological/ cardiovascular disease (81%)  dementia (69%)  Pulmonary disease (38%) | **a)** Baseline assessment including NHO guidelines, documentation, staff interviews  **b)** Identification of criteria for palliative care eligibility  **c)** Noncancer illnesses/conditions  Specific criteria for:  Heart disease, pulmonary disease, dementia, HIV, liver disease, renal disease, acute stroke/coma, chronic stroke | **Adapted NHO guidelines:**   - Surprise question (6 months) - Primary illness/condition with a focus on symptom relief rather than treatment - Several hospitalisations in the last 6 months with a focus on palliation rather than cure - Documentation that the resident’s illness is deteriorating/progressing - Documentation that the resident has reduced nutrition due to their condition - Unexpected crisis that involved a conversation about EOL   **Residents who died:**   - WKPS: mean of 19.4 (p=0.00) - Barthel Index: mean of 2.3 (p = 0.577) - Unplanned weight loss of more than 10% in the past 6 months   **Overall symptoms (moderate or severe) of all residents:**  Weakness/exhaustion (49%)  Restless (42%)  Anxious (42%)  Anorexia (38%)  Constipation (34%)  Oral problems (33%)  Difficulty swallowing (30%)  Rash (26%)  Difficulty breathing (25%)  Depression (22%)  Cough (29%)  Difficulty sleeping (16%)  Pain (16%)  Diarrhoea (12%)  Nausea (9%)  Vomiting (9%)  Oedema (4%) | **a)** Data collected by researcher from staff  **b)** Baseline assessment conducted then every 2 weeks for 10 weeks  **c)** Not reported | **a)** Surprise question of 6 months plus one of the other criteria  **b)** After screening, assessed with WKPS, modified Barthel Index, Abbey Pain Scale, Verbal Descriptor Scale (pain) |
| **a)** Hanson et al.  (2008)  **b)** USA  **c)** Last 3 months and last month of life (actual)  **d)** Mixed method design using telephone interviews | **a)** To detail symptoms of residents at end of life and to make comparisons between staff and family scores  **b)** 674 staff and 446 relatives from 230 LTCF discussing 674 residents  **c)** Female (69%)  **Cause of death:**  Dementia (68%)  Difficulty eating and drinking (62%)  Heart disease (40%)  Infection (28%)  **Age at death:** mean of 85.4 years | **a)** Interview of staff and relatives  **b)** To describe end-of-life symptoms  **c)** Non disease specific | **Three months before death:**  Not independent with mobility (>60%)  **One month before death:**  Personal hygiene need (90%)  Reduced appetite/low intake of fluids (72%)  Anorexia (65%)  Difficulty breathing (48%)  Pain (47%)  Weight loss (32%)  Reduced level of consciousness (29%)  Pressure ulcer (22%)  Choking (16%)  Nausea (8%)  Overall mean symptom severity score of 18.3 (scored 0 to 36, higher=worse)  **Surprise question:**  A week to 2 weeks (15%)  Greater than 28 days (15%)  15-28 days (4%) | **a)** LTCF staff (nurses, HCA, administration) and relatives,  **b)** Not reported  **c)** N/A | **a)** N/A  **b)** Symptom assessment and management, medication management |
| **a)** Hermans et al.  (2017)  **b)** Belgium  **c)** Last 6 months to 1 year of life (predicted)  **d)** Quantitative cross-sectional study | **a)** To detail palliative care needs and symptoms of residents predicted to be in their last year of life and compare differences between dementia and non-dementia residents  **b)** 109 residents from 15 NH  **c)** Average age of 87 years  Female (60%) | **a)** Surprise question (6 to 12 months) and POS  **b)** To describe resident’s palliative care needs and symptoms  **c)** Residents with dementia compared to residents without dementia | - Surprise question (6 to 12 months)   **Overall symptoms based on POS:**  Pain (50%)  Other physical symptom (43%) [2% being an overwhelming problem]  Lacking self-worth (90%)  Requires support (76%)  Anxiety (61%)  More information (38%) [19% being an overwhelming problem]  Difficulty expressing feelings (28%) [28% being an overwhelming problem]  Having a desire to live* (proxy assessment) (13%)  Personal matters (12%)  **Dementia:**  Required more support (p = 0.015)  Had less of a desire to live (p = 0.020)  Had less self-worth (p = 0.12) | **a)** Nurses and nurse assistants  **b)** Not reported  **c)** Not reported | **a)** Eligibility for the study was based on a positive surprise question (6 to 12 months)  **b)** Not reported |
| **a)** Hockley et al.  (2010)  **b)** Scotland  **c)** Last days, weeks, months, or years (predicted)  **d)** Mixed-method design | **a)** “This paper is part of a larger project reporting the impact of implementing both end-of-life care tools together using the same facilitator while proactively visiting the nursing homes two to three times a month using a model of empowerment. The extent to which the goals of the GSF are met are explored.” p.829  **b)** 7 NH and 228 decedents  **c)** Aged 66-104 years  Dementia (60%)  **Age at death:** Over a third were aged 90+ | **a)** Gold Standards Framework for Care Homes  **b)** To enhance the quality of PC provision in the NH, to identify residents’ priorities and choices, to prepare and predict residents’ needs, to enhance staff confidence and collaboration, and to reduce hospitalisations  **c)** Non disease specific | Frailty  Require 24 hour nursing care  + GSF indicators (not reported) | **a)** NH staff  **b)** Monthly discussion  **c)** Intervention implemented over 18 months | **a)** Residents coded according to expected life expectancy:  Code A = years  Code B = months  Code C = weeks  Code D = days  **b) Pathway:**  ‘care needs matrix’  DNR, ACP, GSFCH supportive/palliative care register  Full pathway not reported |
| **a)** Jerant et al.  (2006)  **b)** USA  **c)** Not specified  **d)** Quantitative non-blinded prospective comparison trial | **a)** To compare two interventions aimed at enhancing the PC provided to residents in LTCFs  **b)** 58 intense intervention participants and  23 less intense intervention participants  **c)** More intense group:  Mean age of 84.3 years  Females (74%)  Less intense group:  Mean age of 87.2 years  Females (70%) | **a)** PCAL- assessment (PCS, MCS, MSAS, GDI, GDS, MMSE, self-report of ADLs, PPT, SSS, Values History Tool) and PC recommendation, Eligibility for hospice based on the National Hospice Organisation guidelines  Less intense intervention:  Once off assessment and PC recommendation sent to the resident, relatives, main doctor, and LTCF staff  More intense intervention: Assessments and recommendations every 3 months  **b)** To enhance the PC provided to residents in LTCFs  **c)** Non disease specific | **Symptoms:**  Pain  Low energy  Sadness  Drowsiness  Xerostomia  Difficulty concentrating  Difficulty urinating  Worry  Insomnia  Nervous | **a)** Researcher  **b)** Once off assessment and every 3 months  **c)** Assessments took 1 to 1.5 hours | **a)** Not reported  **b)** Various recommendations given related to: ACD, mobility, hearing, vision, dementia/cognition, pain, depression, ‘other’  Hospice referral |
| **a)** Johnston et al.  (2016)  **b)** Australia  **c)** Last 6 months of life (predicted)  **d)** Qualitative study using focus groups | **a)** To evaluate the implementation of a specialist palliative care model on residents, their family members, and staff.  **b)** 40 staff and 17 family members  **c)** N/A | **a)** Needs Rounds  **b)** To identify residents who are expected to die in the next 6 months and to initiate advance care planning  **c)** Non disease specific | - Surprise question (6 months) | **a)** Palliative care nurse practitioner  **b)** Not reported  **c)** Not reported | **a)** Not reported  **b)** Specialist PC referral, anticipatory prescribing, symptom assessment and management, GP referral, case conferences |
| **a)** Koerner et al.  (2021)  **b)** Australia  **c)** Last months, weeks, or days (predicted)  **d)** Qualitative design with interviews embedded in an RCT | **a)** To determine and provide descriptions of the context and mechanisms that assist with implementing Palliative Care Needs Rounds  **b)** 21 staff interviewed (home managers, nurses, team leaders, care assistants, CNS)  **c)** N/A | **a)** Palliative Care Needs Rounds Checklist (screening tool)  **b)** Identifying those with the greatest need and discussing amongst the team  **c)** Non disease specific | - Indicators from checklist by Forbat et al. (2018)   Before meeting, staff think about who may have the greatest need based on:   - Increased hospitalisations - Who has been acutely unwell - Who has had a decline in their condition | **a)** Specialist PC clinician and care home staff  **b)** Monthly  **c)** Meetings are held for 1 hour | **a)** Not reported  **b)** Discussion with family and GP to discuss goals of care, ACP, managing symptoms, and medication review |
| **a)** Koppitz et al.  (2015)  **b)** Switzerland  **c)** Last 2-3 months, last 1-2 months, and last 8 days- 1 month (actual)  **d)** Mixed-method exploratory, retrospective, descriptive design | **a)** To detail symptoms of nursing home residents with dementia in their last months of life.  **b)** 65 residents from 4 NH  **c)** Female (75.4%)  **Age at death:** mean age of 83.71 years  **Length of residence before death:** mean of 3.5 years | **a)** Nursing notes  **b)** To describe symptoms in the last months of life  **c)** Dementia | **Three months before death:**  Difficulty with mobilising (81%)  Pain (71%)  Difficulty eating (62%)  ‘Unusual behaviour’ (agitation, aggression, lack of interest etc.) (62%)  Difficulty sleeping (63%)  Agitation (39%)  Difficulty breathing (29%)  Lack of interest (25%)  Anxiety (22%)  Depression (14%)  Hospital admission (10%)  Pain increased continually until death  **Other:**  Pneumonia  Lack of appetite  UTI  Dehydration  **T1 = last 2-3 months**  **T2 = last 1-2 months**  **T3 = last 8 days- 1 month**  Anxiety:  T1 (19%), T2 (17%), T3 (29%)  Loss of interest:  T1 (12%), T2 (17%), T3 (24%)  Difficulty breathing:  T1 (12%), T2 (20%), T3 (32%)  Difficulty eating:  T1 (48%), T2 (53%), T3 (75%)  Pain:  T1 (64%), T2 (67%), T3 (72%)  Stress:  T1 (17.2%), T2 (Not reported), T3 (21.5%)  Unusual behaviour:  T1 (67.2%), T2 (9.4%), T3 (61.5%)  Mobility:  T1 (85.9%), T2 (79.7%), T3 (84.6%)  Pressure ulcers:  T1 (26.6%)  Depression:  T1 (20.4%), T2 (15.7%), T3 (15.7%)  Difficulty sleeping:  T1 (60%), T2 (73%), T3 (62%)  Agitation:  T1 (41%), T2 (46%), T3 (42%) | **a)** Data collected by researchers from nursing notes  **b)** Not reported  **c)** N/A | **a)** N/A  **b)** Symptom assessment and management |
| **a)** Krishnan et al.  (2015)  **b)** Canada  **c)** Last 6 months of life (actual)  **d)** Quantitative retrospective cohort study | **a)** To identify factors associated with resident hospital deaths and admissions to hospital in residents last 6 months of life.  **b)** 118 deceased residents  **c)** Female (79.7%)  **Cause of death:**  Advanced dementia (49.2%)  Organ failure (18.2%)  Frailty (17.8%)  **Age at death:** mean of 88.83 years  **Length of residence before death:** 6 days to 11.4 years (mean 2.58 years) | **a)** Chart review and Resident Assessment Instrument- Minimum Data Set  **b)** To identify predictors of hospitalisation in the last 6 months and hospital deaths  **c)** Non disease specific | 4+ comorbidities (11%)  3 comorbidities (12.7%)  2 comorbidities (24.6%)  1 comorbidity (31.4%)  **CPS:**  Severe (4-6) (37.3%)  Mild/moderate (37.3%)  **ADLs:**  Moderately dependent (52.5%)  Difficulty with eating (37.3%)  Severely dependent (34.7%)  **Hospital transfers in the last 6 months:**  0 (82.2%)  1+ (17.8%) | **a)** LTC staff  **b)** MDS is completed every 3 months or if there has been a change in the resident’s condition  **c)** N/A | **a)** N/A  **b)** Advance Care Planning |
| **a)** Lawrence, Robinson, and Eagar  (2017)  **b)** Australia  **c)** Last 11 years of life (actual)  **d)** Quantitative retrospective longitudinal study | **a)** To determine diagnostic groups and the deterioration trajectory of nursing home residents with a view to informing advance care planning  **b)** 247 resident’s records from 4 NH  **c)** Mean age of 84 years  Female (69%)  **Age at death:** mean of 86.8 years  **Length of residence before death:** mean of 3.3 years | **a)** Resident Classification Scale (RCS)  **b)** To identify specific groups and predict decline to inform ACP  **c)** Cancer, organ failure, frailty, other | **RCS:**   - Average level of functional performance when admitted was 51.8 which declined to 70.8 pre death - Men admitted with reduced functional performance lived longer (p = 0.005) - Women admitted with reduced functional performance died younger (p = 0.001) - Deterioration of functional performance from admission until death was different for men (18%) and women (29%) - The RCS deteriorated at an average rate of 10.3 annually (p= <0.001)   **Cancer:** Shortest time in the nursing home, functional deterioration of 16% from time of admission until death. Residents with cancer had a deterioration of 19.8 points annually. This raised by 5.5 points in the year preceding death.  **Organ failure:** Longest stay in NH until death. Functional deterioration of 17% from time of admission until death. Had an average RCS of 68.6 when dying (CI 62.4-74.8).  **Frailty:** Mean stay of 3.4 years in the NH until death. Functional deterioration of 21% from time of admission until death. Had the highest RCS when dying (p = <0.001). They had a prolonged and slower deterioration of 9.5 points annually. This raised by 1.2 points in the year preceding death.  **Other:** Best functional performance when admitted. Functional deterioration of 17% from time of admission until death. Lowest in dependency pre death. | **a)** NH staff  **b)** RCS measured on admission and then annually or if indicated (e.g. change in condition)  **c)** Not reported | **a)** RCS (a high score indicates the resident has a large number of care needs, scored 0-100).  Classification into four groups: cancer, organ failure, frailty, and ‘other’  **b)** Not reported |
| **a)** Lima and Miller  (2018)  **b)** USA  **c)** Not specified  **d)** Quantitative descriptive longitudinal study | **a)** To detail characteristics of residents who had a palliative care consultation  **b)** 875 residents from 54 NHs (4% had a PC consult)  **c)** PC residents: Mean age of 80.82 years  Short stay residents:  Females (62.6%)  Cancer without dementia (22%)  Cancer with dementia (9.8%)  Dementia without cancer (32.8%)  Long stay residents:  Females (69.5%)  Cancer without dementia (6.2%), Cancer with dementia (11.5%)  Dementia without cancer (61%) | **a)** Medicare data, RAI-MDS data, nursing notes, ADL score, CPS  **b)** To describe characteristics of residents who had a palliative care consultation  **c)** Non disease specific | **Short stay residents (less than 90 days):**  Unstable condition (79.5%) (p<0.001)  Acute events (70%) (p<0.001)  Pain every day (34.9%)  Weight loss (13.1%)  CPS score 5+ (6.2%)  Prognosis of less than 6 months (3.2%) (p<0.05)  ADL mean 17.69 (p<0.001)  **Long stay residents (more than 90 days):**  Unstable condition (64.3%) (p<0.001)  Acute events (26.6%) (p<0.001)  Pain every day (15.7%)  Weight loss (14.2%) (p<0.05)  CPS score 5+ (12.5%)  Prognosis of less than 6 months (2%) (p<0.05)  ADL mean 17.27 (p<0.001) | **a)** Nursing home staff  **b)** Not reported  **c)** N/A | **a)** N/A  **b)** PC referral, hospice referral, DNR, do-not-hospitalise order |
| **a)** Liu et al.  (2020)  **b)** Australia  **c)** “Last months of life” (predicted)  **d)** Quantitative step wedged RCT | **a)** To evaluate the effect that specialist palliative care has on the quality of death and dying for nursing home residents  **b)** 563 out of 1700 residents from 12 care homes  **c)** Mean age of 85 years  Females (64%)  Dementia (30%)  CVD (14%)  Frailty (8%)  **Age at death:** Mean of 86 years | **a)** Palliative Care Needs Rounds Checklist (screening tool)  **b)** To discuss residents who have been identified as likely to die and do not have a plan  **c)** Non disease specific | - Surprise question (6 months) - Physical/ cognitive deterioration in previous month - Lack of ACP or plan for last 6 months before death - Disagreements amongst the resident relatives around treatment/care - A direct transfer for EOL care | **a)** Specialist palliative care professionals (nurse practitioners and clinical nurse consultant) and care home staff (nurses, HCAs, managers, activities coordinators)  **b)** Monthly  **c)** 1 hour long meetings | **a)** Resident must have 1+ of the indicators to be discussed  **b)**  **1.** Identify diagnosis and comorbidities  **2.** Identify palliative care needs  **3.** Identify staff concerns  **4.** Identify residents support structure  **5.** Educate staff on potential scenarios specific to that resident (e.g. recognising deterioration and dying)  **6.** Review medications  **7.** Ensure resident has a proxy decision maker if necessary  **8.** Consider ACP  **9.** Consider planning a case conference  **10.** Consider outside referrals (e.g. pastoral care, wound care)  **11.** Consider specialist palliative care referral  **As well as:** Symptom management, anticipatory prescribing |
| **a)** Liyanage, Mitchell, and Senior  (2018)  **b)** Australia  **c)** Last year of life (predicted)  **d)** Quantitative prospective cohort study | **a)** To identify the accuracy, feasibility, and acceptability of using the SQ with SPICT to identify residents with palliative care needs  **b)** 187 residents from 2 RACFs  **c)** Mean of 82.39 years  Female (55.2%) | **a)** Surprise question (12 months) together with SPICT (screening tool)  **b)** Identification of residents with palliative care needs  **c)** SPICT clinical indicators: cancer, dementia/frailty, neurological disease, cardiovascular disease, respiratory disease, kidney disease, and liver disease. | - Surprise question (12 months)   **General indicators:**   - Performance status decline - 2+ unexpected hospital admissions in 6 months - 5-10% weight loss in 3-6 months or BMI below 20 - Consistent bothersome symptoms even with treatment - Dependent for majority of care needs [this replaced an older indicator in 2015 (resident of a RACF)] - Residents asks for PC or cessation of treatment   **Disease specific:**  Cancer  Functional capacity declining related to metastasis  Frailty prevents person from receiving curative treatment or treatment for symptom management  Dementia/frailty  Inability to dress, eat, or ambulate independently  Reduced appetite, imbalanced nutrition  Incontinence  Inability to communicate, social withdrawal  Femoral fracture, recurrent falls  Repeated episodes of pyrexia, infection, or aspiration pneumonia  Neurological disease  Consistent decline in physical or cognitive functioning regardless of treatment  Difficulty with speech or swallowing  Aspiration pneumonia, difficulty breathing, respiratory failure  Cardiovascular disease  NYHA stage III/IV HF, CAD  Severe peripheral vascular disease  Respiratory disease  COPD, pulmonary fibrosis  Oxygen therapy over a long period  Required ventilation as a result of respiratory failure  Kidney disease  CKD  Kidney failure (other cause)  Discontinuing dialysis  Liver disease  Liver cirrhosis  Contraindication for a liver transplant  “1 or more clinical indicator” | **a)** Directors of nursing created a list of residents in the study  **b)** Not reported  **c)** Average of 18.6 seconds | **a)** Must have positive SQ to move on to assessment with SPICT.  **SPICT:** At least 2 general indicators of declining health and 1 clinical indicator.  **b)** Specialist PC consult or advice, GP consult, PC nurse specialist consult, ACD, ACP, consultation with family, staff, and clinician |
| **a)** Mitchell et al.  (2004)  **b)** USA  **c)** Last 6 months of life (actual)  **d)** Quantitative retrospective cohort study | **a)** To outline and make comparisons of residents with advanced dementia’s experience at end of life.  **b)** 2730 residents  **c)** Mean age of 83.4 years  Female (63%)  CHF (29.6%)  Chronic Pulmonary Disease (15.6%)  Cancer (11.4%)  **Age at death:** mean of 83.4 years  **Length of residence before death:** all residents died within 1 year | **a)** MDS-NH and  MDS ADL scale  **b)** To identify EOL symptoms, experiences  **c)** Dementia | **Residents with dementia:**  Older (mean age of 83.4 years) (p 0.01)  Shorter length of stay (mean of 173.7 days) (p <0.001)  More dependent (39.3% had ADL score of 28) (p <0.001)  CPS score of 6 (57.1%) (p <0.001)  Hospitalisations in the 3 months before death (43.7%)  Behaviour issues (31.1%) (p <0.001)  Surprise question of less than 6 months in the 6 months before death (10.3%)  **Symptoms:**  Pain (37.1%)  Pressure ulcers (35%)  Catheterised (29.4%)  Receiving oxygen (24.4%)  Depression (20.5%)  Pneumonia (16.9%)  Difficulty breathing (12.7%) | **a)** Data collected by nursing home staff  **b)** Completed at admission, once a year and if there has been a substantial change to the resident’s health  **c)** Not reported | **a)** MDS ADL Scale (scored 0-28. Score of 28 = fully dependent)  **b)** Hospice referral, ACP, ACD, DNR, Do Not Hospitalise order, medication therapy, pain management |
| **a)** Mitchell, Kiely, and Hamel  (2004)  **b)** USA  **c)** Last 3 months of life (actual)  **d)** Quantitative design | **a)** To describe and compare the end of life experiences of residents with advanced dementia and residents with terminal cancer  **b)** 1609 residents with advanced dementia and 883 residents with terminal cancer  **c)** Mean age of 81.3 years  Dementia (64%)  Females (57.1%)  Cancer (36%)  Females (52.1%)  **Age at death (mean):**  Dementia (83.5) Cancer (79.1)  **Length of residence before death (mean):**  Dementia (121.6 days)  Cancer (62.4 days) | **a)** Minimum Data Set  **b)** To describe EOL symptoms, characteristics of residents  **c)** Advanced dementia compared with terminal cancer | **Three months before death:**  1.1% of those with dementia had positive surprise question (6 months) but 71% died within those 6 months  **More common in residents with cancer:**  Pain: dementia (11.5%), cancer (56.6%) (p <.001)  Constipation: dementia (13.7%), cancer (32.7%) (p <.001)  Difficulty breathing: dementia (8.2%), cancer (27.6%) (p <.001)  **More common in residents with dementia:**  Difficulty eating/swallowing: dementia (45.9%), cancer (33.6%) (p <.001)  Weight loss: dementia (26.1%), cancer (41.7%) (p <.001)  Pressure ulcers: dementia (14.7%), cancer (6%) (p <.001)  Pyrexia: dementia (13.4%), cancer (6.8%) (p <.001)  Pneumonia: dementia (10.8%), cancer (3.6%) (p <.001)  **Other:**  Depression: dementia (9.1%), cancer (12.7%)  Anxiety: dementia (2.8%), cancer (5%)  Hallucinations: dementia (2%), cancer (5.1%)  Mean ADL score: dementia (23.3), cancer (18.3) (p <.001)  Mean BMI: dementia (29.1), cancer (32.6) | **a)** Data collected by nursing home staff  **b)** Assessed in first 2 weeks after admission and then assessed every 3 months  **c)** Not reported | **a)** N/A  **b)** ACP, ACD, documenting resuscitation status, documenting treatment and hospitalisation preferences, palliative care, pain management |
| **a)** Mitchell et al.  (2011)  **b)** Australia  **c)** Various ‘phases’ according to PCOC criteria. Time frame only given to terminal phase (days to live) (actual)  **d)** Mixed-method cross-sectional study using surveys and focus groups | **a)** To identify the resident’s needs and quality of palliative care and to identify staff’s needs to deliver palliative care  **b)** 27 family members and 28 healthcare workers (2 GPs, 7 registered nurses,15 enrolled nurses or care assistants, 2 ancillary staff, 1 counsellor, 2 community palliative care nurses) from 16 facilities  **c)** Aged 80+ (78%)  Female (67.1%)  Mental and behavioural disorders (including dementia) (28.7%)  Cardiovascular diseases (13.7%)  Cerebrovascular diseases (8%) | **a)** PCOC used to identify the phase the residents were at (stable, unstable, deteriorating, or terminal), AKPS and PSS also used  **b)** To identify residents PCOC phase  **c)** Non disease specific | PCOC criteria not listed  AKPS  Unstable residents (3.9%)  Declining or terminal residents (3.2%)  Unstable residents had more functional impairment (p = <0.001)  Unstable residents had more hospital admissions in previous 3 months than the stable or declining/terminal residents (p = <0.001) | **a)** Healthcare workers  **b)** Not reported  **c)** Not reported | **a)** Residents classified as stable, unstable, deteriorating, or terminal according to PCOC (no further elaboration)  **b)** ACP, pain management, aromatherapy, music, support to relatives, specialist PC referral |
| **a)** Phillips et al.  (2008)  **b)** Australia  **c)** Not specified  **d)** Qualitative study on a 3 year participatory action research project | **a)** To identify the perceptions of nursing home staff on a palliative care intervention  **b)** 28 nurses, care assistants, and directors of nursing from 9 RACF  **c)** N/A | **a)** Intervention involved implementing a link-nurse, educating staff and GPs, and providing the opportunity for advice from palliative care experts  **b)** To improve staff PC education, and to enhance their connections to PC specialists  **c)** Non disease specific | Not reported.  Assessment tools found to be useful by staff (no specific tools named) | **a)** Care home staff (nurses, carers) and link nurse  **b)** Not reported  **c)** Not reported | **a)** Not reported  **b)** Specialist PC referral, pain assessment and management |
| **a)** Porock et al.  (2003)  **b)** USA  **c)** Last 6 months of life (predicted)  **d)** Quantitative descriptive, correlational retrospective cohort design | **a)** To detail the prevalence, profile, and survivorship of long term care residents  **b)** 9615 residents, 432 (4.5%) designated as EOL from 512 LTCF  **c)** Female (60%)  Cancer (56%)  Emphysema/COPD (24%)  CHF (20%)  Alzheimer’s/Dementia (2%)  **Length of residence before death:** median of 33 days | **a)** Minimum Data Set (MDS) designations:  End-stage disease- 6 or fewer months to live or Hospice care  **b)** To describe characteristics of EOL residents  **c)** Non disease specific | Chemotherapy (OR 7.79)  Pain- severe to excruciating (OR 6.98)  DNR (OR 5.52)  Do not hospitalise (OR 5.50)  Pain- moderate to severe (OR 4.37)  Oxygen therapy (OR 3.71)  Dialysis (OR 3.52)  Radiation (OR 3.49)  Insufficient fluid (OR 3.15)  Dehydrated (OR 2.98)  Weight loss (OR 2.77)  Skin ulcer (OR 2.24)  Parenteral/IV (OR 2.16)  Depression (OR 1.66)  Incontinence (OR 1.30) | **a)** NH staff  **b)** Completed within 14 days of being admitted, every year, and if there is a considerable change in the resident’s condition,  **c)** Not reported | **a)** Not reported  **b)** Hospice referral, Advance Care Directive |
| **a)** Pruitt  (2022)  **b)** USA  **c)** Not specified  **d)** Dissertation with a prospective, descriptive design | **a)** To improve identification of symptoms and increase palliative care referrals for nursing home residents  **b)** 52 out of 180 residents from 1 NH (11 referred for PC)  **c)** Mean age of 72.9 years  Female (53.8%) | **a)** The Palliative Care Screening for the Elderly tool (screening tool)  **b)** To improve identification of symptoms of people with dementia and referrals to palliative care  **c)** Dementia | Reduced oral intake for 3+ days  Weight loss of more than 5% in last 3 months  Incontinence  Reduced physical activity (reduced independence with ADLs)  New or worsening pressure ulcer  Fall in the past 30 days with severe harm  Went to the hospital or ED in the past 30 days  **Associations with referrals:**  Reduced physical activity (p <0.001)  ED admission (p 0.02)  Reduced oral intake (p 0.034)  Falls + severe harm (p 0.041) | **a)** Nurse assistant, nurse, cleaners, activity coordinator  **b)** Monthly  **c)** ‘Minimal time’ | **a)** Must have at least 3 of the indicators for a referral,  However, the tool states if yes to ‘any’ then escalate to ADON  **b)** Palliative Care referral |
| **a)** Puente-Fernández et al.  (2020)  **b)** Spain  **c)** Last 6 months of life (predicted)  **d)** Quantitative prospective cross sectional study | **a)** To detail symptoms, palliative care outcomes, medications, tests, and procedures of palliative care residents and to make comparisons between those with and without dementia  **b)** 107 residents from 6 NH  **c)** Mean age of 84 years  Female (68%)  Arterial hypertension (58.9%)  Vascular/brain damage (42.1%)  Diabetes (31.8%)  HF (26.2%) | **a)** Spanish Society of Palliative Care (SECPAL) criteria, ESAS and POS also used to assess  **b)** To identify residents as palliative and to describe their symptoms  **c)** People with dementia compared to people without dementia | **SECPAL criteria:**  Advanced, life limiting illness  Low expectation to respond to treatment  Complex symptom and many challenges  Emotional effect on resident   - Prognosis of 6 months or less   **Symptom intensity for both groups:**  Pain (87.9%)  Drowsiness (86%)  Fatigue (82.2%)  Feeling unwell (74.8%)  Lack of appetite (68.2%)  Difficulty sleeping (64.5%)  Anxiety (62.6%)  Depression (61.7%)  Difficulty breathing (54.2%)  Nausea (33.6%)  **Dementia:**  Dyspnoea- most intense symptom  Drowsiness- second most intense symptom  POS- mean of 1-2  More dependent (p = 0.001)  **Non-dementia:**  Fatigue- most intense symptom  Depression- second most intense symptom  COPD- more prevalent  POS- mean of 2-3  Higher anxiety (p= 0.05)  Higher depression (p = 0.05) | **a)** Two trained nurses  **b)** Not reported  **c)** Not reported | **a)** Resident must meet all of SECPAL criteria  **b)** Not reported |
| **a)** Reynolds et al.  (2002)  **b)** USA  **c)** Last 3 months of life (actual)  **d)** Qualitative interviews | **a)** To detail palliative care needs of residents in their 3 months before death  **b)** 176 nurses, care assistants, and relatives from 2 NH (discussing 80 deceased residents)  **c)** Mean age of 82 years  Female (61%)  Cause of death:  Pneumonia (19%)  CHF/CAD (19%)  Cancer (17%)  **Age at death:** mean of 82 years  **Length of residence before death:** 39% of residents resided in the NH for longer than a year | **a)** Staff assessment  **b)** To describe resident’s palliative care needs  **c)** Non disease specific | **Three months before death:**  Pain (86%)  Personal hygiene need (81%)  Required help with dressing (81%)  Required help with mobilising (79%)  DNR (79%)  Difficulty breathing (75%)  Wanted comfort care (70%)  Incontinence (59%)  Hospital admission (58%)  Fatigue (52%)  Death anticipated (51%)  Required help with eating (49%)  Depression (44%)  Anxiety (31%)  Orders to stop other treatments and not to hospitalise (39%)  Felt lonely (21%)  Experienced constipation, choking, nausea, difficulty sleeping, and anorexia (15-21%)  Cognitive issues (14-59%)  Ventilated (10%)  Had CPR performed (8%)  Required more support emotionally/spiritually (30%)  Required more support with personal hygiene (23%)  Required more interventions for pain (19%) | **a)** Nurses, care assistants, relatives  **b)** Not reported  **c)** N/A | **a)** N/A  **b)** Hospice referral, pain management, documentation of treatment preferences |
| **a)** Robinson et al.,  (2020)  **b)** New Zealand  **c)** Last year of life (actual)  **d)** Quantitative cross sectional design | **a)** To examine the use of 2 InterRAI tools to determine occasions to initiate palliative care in long term care facilities  **b)** 1528 people (had either Home Care or LTC assessments)  613 = LTC  **c)** Aged 80+ (68.1%)  Female (52.5%)  Dementia (43.8%)  Cardiovascular disease (38.3%)  Neurological condition (35.3%)  Cancer (26.5%) | **a)** International Resident Assessment Instrument (InterRAI)  **b)** To identify occasions to initiate palliative care  **c)** Non disease specific | DNR documentation (72.8%)  Documentation of prognosis of less than 6 months (15.6%) | **a)** LTCF staff  **b)** Not reported  **c)** Not reported | **a)** N/A  **b)** To prompt palliative care interventions,  Advance Care Planning- documenting resuscitation status and residents’ preferences regarding treatment/ hospitalisation |
| **a)** Schmidt et al.  (2018)  **b)** Germany  **c)** “Final phase of life” (actual)  **d)** Qualitative multi perspective study using grounded theory | **a)** To describe the needs of residents with advanced dementia in their last phase of life  **b)** 42 HCP (carers, doctors, cleaners, “other”) from 6 NH and 14 family members  **c)** N/A | **a)** Semi structured group discussions and interviews  **b)** To identify end of life needs  **c)** Dementia (residents had to be stage 6 or 7 of GDS and unable to communicate verbally) | **Physical**  Difficulty with eating + drinking  Self-care ability/need for assistance  Oral health  Excretion  Dressing  Pain  Distress related to disease e.g. infection  Sensory stimuli- positive effects  Mobility  Rest and sleep  **Psychosocial**  Overstimulated  Under stimulated  Convey emotions  Socialising  Being comprehended  Physical contact with others  Experiencing daily activities  Involvement in activity  Having familiar surroundings  Feeling secure  Conveying their wishes  Being themselves  **Spiritual**  Conveying religion  Partaking in religious ceremonies | **a)** Healthcare professionals and family members  **b)** Not reported  **c)** N/A | **a)** N/A  **b)** Protect from stimuli e.g. stimuli that may be overwhelming (such as too much light), provide stimuli e.g. stimuli that promotes their wellbeing |
| **a)** Strumpf et al.  (2004)  **b)** USA  **c)** Not reported  **d)** Narrative article discussing an interventional study | **a)** The aim of the intervention was to improve overall PC provision in the NHs including ACP, symptom assessment and management, and supporting residents psychological and social needs  **b)** 6 nursing homes- 4 receiving intervention, 2 as the control  **c)** Average age of 78 years  Females (68%) | **a)** Assessment and screening tools (not elaborated on),  **b)** To identify symptoms and needs  **c)** Non disease specific | **Eligibility criteria:**   - Terminal or end-stage disease - Substantial functional deterioration - Several hospital admissions recently - Change in nutritional status   **Symptoms:**  Pain (77%)  Constipation (77%)  Weakness (71%)  Cough (46%)  Depression (36%)  Anxiety (24%)  **Other (no % reported):**  Nausea  Difficulty breathing  Anorexia  Spiritual needs | **a)** Nurse consultant helped nurses and social workers to identify residents who may be suitable for palliative care  **b)** When admitted and then every 3 months, (although those identified as potential for PC were discussed at weekly meetings)  **c)** Not reported | **a)** Any 1 of the eligibility criteria  **b)** Advance Care Planning, symptom assessment and management |
| **a)** Todd et al.  (2021)  **b)** UK  **c)** Last 6 months of life (predicted), however data collected over 12 months  **d)** Quantitative design using questionnaires | **a)** To describe end of life needs of residents with intellectual disability  **b)** 132 people (22 died) with intellectual disabilities from 66 LTCF  **c)** Mean age of 68.8 years  Epilepsy (27.6%)  Diabetes (18.9%)  Arthritis (18.4%)  Dementia (17.6%)  **Age at death:** mean of 72.2 years | **a)** Surprise question (6 months) and questionnaires  **b)** To identify end of life needs  **c)** Residents with intellectual disabilities | Surprise question of ‘no’ for 23.3% of residents (40.7% of those died p <0.01)  Sensitivity (55%)  Specificity (84.5%) | **a)** A manager or senior nurse  **b)** Not reported  **c)** Not reported | **a)** N/A  **b)** Not reported |
| **a)** Tuch, Parish, and Romer  (2003)  **b)** USA  **c)** Not specified but study conducted over 3 years  **d)** Interview of authors who conducted an intervention study over 3 years comparing 2 interventions | **a)** To implement a model of palliative care into care homes  **b)** 553 residents from 4 intervention NH and not reported number of residents from 2 control NH  **c)** Not reported | **a)** **1:** Staff education alone  **2:** Staff education plus the establishment of a PC team (Staff education and PC team meetings)  **b)** To incorporate specialist PC knowledge and procedures into care homes  **c)** Non disease specific | “Palliative Care Screen” screens for:   - advanced cancer - life-limiting or terminal-stage disease (diseases such as cardiovascular disease, dementia, COPD included) - Relatives and residents’ concerns | **a)** Care home staff (dietitians, nurses, social workers, activities manager, care home staff, palliative care co coordinators)  **b)** Weekly team meetings, relatives/residents also invited  **c)** 45 minute meetings | **a)** Unclear, staff refer to the PC coordinator based on “Palliative Care Screen", then NHO guidelines used, then “Steps” checklist  **b)** **1.** Referral to the palliative care coordinator or nurse completing admission for a more in depth assessment  **2.** Then tool based on the National Hospice Organization guidelines used  **3.** If deemed eligible at this point resident moves on to “Steps to Implement the Palliative Care Delivery Process” checklist (identifies tools to be used)  **As well as:** Hospice referral, PC referral, symptom assessment and management, ACP |
| **a)** Vandervoort et al. (2013)  **b)** Belgium  **c)** Last month of life (actual)  **d)** Quantitative retrospective cross-sectional study using questionnaires | **a)** To explore the number of residents with dementia who died and to detail their characteristics and quality of dying  **b)** 198 residents from 69 NH  **c)** Mean age of 86.7 years  Female (61.5%)  Cardiovascular disease (28.8%)  Respiratory disease (13.5%)  Neurological disease (15.3%)  **Age at death:** mean of 86.7 years  **Length of residence before death:** median of 2.4 years | **a)** CPS, GDS, BANS-S, SM-EOLD, MMSE  **b)** To describe characteristics of deceased residents  **c)** Dementia | **One month before death:**  Mean BANS-S of 20.8  95.5% had 1+ sentinel event (dysphagia (65.7%), pyrexia (42.9%), pneumonia (32.3%))  Dysphagia increases with severity of dementia (p = .03)  MMSE of less than or equal to 10 (58.1%)  GDS Stage 7 (57.7%)  CPS Score 6 (45.9%)  **Symptoms from SM-EOLD scale:**  Pain (mean 1.9)  Fear (mean 2.3)  Anxiety (mean 2.4)  Agitation (mean 2.9)  Opposing care (mean 3.0)  Difficulty breathing (mean 3.2)  Had a co morbidity (70.3%) | **a)** Data collected by NH staff (nurses, doctor, nursing admin)  **b)** Not reported  **c)** Not reported | **a)** SM-EOLD scale (scored 0-5. Lower score = more symptoms)  **b)** Symptom assessment and management |

**Data extraction table 1b: Review and expert opinion**

| **Paper Details** | | | **Paper Characteristics** | | **Assessment and Implementation** |
| --- | --- | --- | --- | --- | --- |
| **a) Reference**  **b) Country**  **c) Time frame** | **a) Design**  **b) Sample size** | **Aim** | **a) Method**  **b) Assessor**  **c) Specificity to disease** | **Indicators** | **a) Recommended frequency of assessment**  **b) Referral pathway** |
| **a)** Cole at al.  (2023)  **b)** USA  **c)** Not specified | **a)** Systematic review  **b)** Two included papers | To determine validated palliative care screening tools used in nursing homes and to appraise and summarise such tools critically | **a)** NECPAL identified as the only screening tool meeting COSMIN criteria  **b)** Doctors, nurses, social workers, psychologists, experts in palliative care  **c)** NECPAL disease specific criteria: cancer, COPD, chronic heart disease, liver diease, renal disease, stroke, dementia, neurodegenerative diseases, AIDS, other. | **NECPAL criteria:**   - Surprise question+ (12 months) plus 1 of: - Resident, relatives, or HCP expresss need for PC - Karnofsky/Barthel <30% - Loss of 2+ ADLs - Karnofsky <50 - Barthel <20 - >3 Pfeiffer (cognitive delcline) - Weight loss greater than 10% in the previous 6 months - 2+ unplanned hospitalisations in the previous 6 months - 2+ of: falls, difficulty swallowing, pressure ulcers, delirium, recurring infections - 2+ of: pain, weakness, lack of appetite, digestive dysfunction - Emotional distress, social isolation - Plus disease specific indicators | **a)** Not reported  **b)** Not reported (NECPAL does have its own pathway however) |
| **a)** Ersek and Carpenter  (2013)  **b)** USA  **c)** Not specified | **a)** Literature review  **b)** Number of included studies not reported | To examine the available literature on palliative care in long-term care and to determine gaps in the literature and make recommendations for future research | **a)** Minimum Data Set, reviewing charts, healthcare professional identification, relative identification, interviewing the residents themselves  **b)** LTC staff, relatives, doctors, residents  **c)** Non disease specific | Pain  Difficulty breathing  Difficulty eating  Delirium  Incontinence  Noisy respirations | **a)** Not reported  **b)** ACD, ACP, hospice referral, DNR, POLST, symptom management |
| **a)** Evans  (2009)  **b)** UK  **c)** Last year of life (predicted) | **a)** Literature review, expert opinion, and retrospective analysis  **b)** 40 residents in 1 care home | To discuss various EOL topics specific to residents with dementia such as: ACP, GSF-PIG, pain assessment, care at EOL, emergencies, hospitalisations, and dysphagia | **a)** Modified Gold Standards Framework- Proactive Identification Guidance (GSF-PIG), expert opinion  **b)** GP, consultants, nurses, SALT, psychologist, dietitian  **c)** Dementia | **Modified GSF-PIG criteria** (scored 0-150. Score of 150 = most independent and healthy. Score below 75 = more likely to die):   - Meaningful oral communication (+10) - Lower than 10% weight loss in the last 6 months (+10) - Absence of pressure sores (+10) - Absence of aspiration pneumonia (+10) - Absence of repeated urinary tract infections or pyleonephritis (+10) - Barthel score (0-100)   **Other:**  Dysphagia | **a)** Annually  **b)** Advance care planning- wishes of resident/family, care they wish to receive or not receive, preference of place of death, medication review, DNR (“allow a natural death form”) |

**Abbreviations:**

ACD: Advance Care Directive, ACH: Aged Care Home, ACP: Advance Care Planning, ADL: Activity of Daily Living, ADON: Assistant Director of Nursing, AKPS: Australian-modified Karnofsky Performance Scale, AIDS: Acquired Immunodeficiency Syndrome, ALS: Amyotrophic Lateral Sclerosis, BANS-S: The Bedford Alzheimer's Nursing Severity-Subscale, BMI: Body Mass Index, CaCI: Case Complexity Index, CAD: Coronary Artery Disease, CFS: Clinical Frailty Scale, CHD: Coronary Heart Disease, CHESS: Changes in Health, End stage disease, and Symptoms and Signs, CHF: Congestive Heart Failure, CKD: Chronic Kidney Disease, CNS: Clinical Nurse Specialist, COPD: Chronic Obstructive Pulmonary Disease, CPR: Cardiopulmonary Resuscitation, CPS: Cognitive Performance Scale, CVA: Cerebrovascular Accident, DNR: Do Not Resuscitate, ED: Emergency Department, EHR: Electronic Health Record, EOL: End of Life, Frail VIG Index: VIG is Spanish abbreviation for CGA (Comprehensive Geriatric Assessment), ESAS: Edmonton Symptom Assessment Scale, GDI: General Distress Index, GDS: Geriatric Depression Scale, GDS: Global Deterioration Scale, GP: General Practitioner, GSF: Gold Standards Framework, GSFCH: Gold Standards Framework for Care Homes, GSF-PIG: Gold Standards Framework Prognostic Indicator Guidance, HC: Historical Control, HCA: Health Care Assistant, HCP: Healthcare Professional, HF: Heart Failure, HIV: Human Immunodeficiency Virus, IDCPal: Diagnostic Instrument of Complexity in Palliative Care, ID: Intellectual Disability, IR: Intervention Residents, IV: Intravenous, LTC: Long Term Care, LTCF: Long Term Care Facility, MCS: Mental Component Scale, MDS: Minimum Data Set, MMSE: Mini Mental State Exam, MND: Motor Neuron Disease, MS: Multiple Sclerosis, MSAS: Memorial Symptom Assessment Scale, N/A: Not Applicable, NH: Nursing Home, NHO: National Hospice Organization, NECPAL: Necesidades Paliativas, NYHA: New York Heart Association, OR: Odds Ratio, PC: Palliative Care, PCOC: Palliative Care Outcomes Collaboration, PCS: Physical Component Scale, POS: Palliative Care Outcome Scale, POLST: Physician Orders for Life-Sustaining Treatment, PPS: Palliative Performance Scale, PPT: Physical Performance Test, PSS: Palliative Care Problem Severity Score, RACF: Residential Aged Care Facility, RAI: Resident Assessment Instrument, RCS: Resident Classification Scale, RCT: Randomised Control Trial, RN: Registered Nurse, SALT: Speech and Language Therapist, SM-EOLD: Symptom Management End of Life in Dementia, SPICT: Supportive and Palliative Care Indicator Tool, SQ: Surprise Question, SSS: Social Support Survey, UK: United Kingdom, USA: United States of America, UTI: Urinary Tract Infection, WKPS: WARP Karnofsky Performance Scale
