# Supplementary material for: Identifying unmet palliative care needs of nursing home residents: A scoping review
Source: PLoS One. 2025 Feb 25;20(2):e0319403. doi: 10.1371/journal.pone.0319403 (PMC11856323; doi:10.1371/journal.pone.0319403)
Supplement: S2 Table — (DOCX) [file pone.0319403.s004.docx]

**Data extraction table 2a: Guidelines**

| **Document Details** | | | **Document Characteristics** | | **Assessment and Implementation** |
| --- | --- | --- | --- | --- | --- |
| **a) Reference**  **b) Country**  **c) Time frame** | **Document name** | **Document aim** | **a) Type of document**  **b) Intended users**  **c) Specificity to disease** | **Indicators** | **a) Recommended frequency of assessment**  **b) Referral pathway** |
| **a)** National Health Service  (2010)  **b)** UK  **c)** Dying in the “near future” (predicted) | “The route to success in end of life care - achieving quality in care homes” | It is a part of the Department of Health’s End of Life Care Strategy (2008), aiming to improve the quality of care for those who are dying and their relatives. | **a)** Guide  **b)** Care home staff and managers  **c)** Non disease specific | Surprise Question (“near future”)  Significant life event such as death of husband/wife  More frequent hospitalisations | **a)** “ongoing basis” p. 7  **b)** **1.** Identify triggers and start discussion with team, resident, relatives  **2.** Complete full needs and preferences assessment, update care plan, and review ‘regularly’  **3.** Implement care plan, liaise with external services  **4.** Create a suitable environment, ensure resident is cared for with dignity, use external services if needed, use resources efficiently  **5.** Care of the resident in their last days  **6.** Care after death  **As well as:** ACP, ACD, family meetings, holistic needs assessment and management, anticipatory medication prescribing, GP referral, specialist PC referral, creating an appropriate environment, spiritual services, and educating resident and relatives. |
| **a)** The National Palliative Care Program  (2006)  **b)** Australia  **c)** Not specified | “Guidelines for a Palliative Approach in Residential Aged Care” | Aims to support and guide staff in Residential Aged Care Facilities (RACF) in the provision of palliative care. | **a)** Guideline  **b)** RACF staff  **c)** Non disease specific but has section specific for advanced dementia | **Indicators:**  Pain  Fatigue  Weight loss  Depression  Cachexia  Dysphagia  Impaired skin integrity  Constipation  Difficulty breathing  Anxiety  Delirium  Personal hygiene  Incontinence  Loneliness  Resident/relatives opting for ‘symptom relief’ over ‘curative care’  Reduced functional ability with ADLs  Lack of appetite  Mucositis  Difficulty sleeping  Nausea and vomiting  Dehydration  Confusion  Expression of spirituality  Engage in spiritual practices  Emotional distress  Emotional preparation  **Advanced dementia signs:**  Confusion  Incontinent of urine  Pain  Depression  Constipation  Loss of appetite  Dysphagia | **a)** ‘Regular basis’  **b)** Symptom assessment and management, complimentary therapies, family meeting, resident and family education/support, timely referral to specialist PC, medication management, anticipatory prescribing, chaplain/pastoral care referral, provide emotional support, spiritual services or counselling, identify spiritual beliefs |

**Data extraction table 2b: Papers reporting on guidelines**

| **Paper Details** | | | **Guideline Characteristics** | | | **Assessment and Implementation** |
| --- | --- | --- | --- | --- | --- | --- |
| **a) Reference**  **b) Country**  **c) Time frame** | **a) Guideline name**  **b) Type of paper** | **Paper aim** | **a) Intended users**  **b) Specificity to disease** | **Indicators** | | **a) Recommended frequency of assessment**  **b) Referral pathway** |
| **a)** Gill et al.  (2011)  **b)** Canada  **c)** Not specified | **a)** “The nursing guidelines for EOL care in LTC settings”  **b)** Mixed-Method design used to evaluate guidelines (surveys and interviews) in 2 LTC homes | “This paper describes the results of the pilot project on the development and implementation of nursing guidelines for EOL in LTC settings and the impact of guidelines on the management of EOL care within the participating LTC homes; it also highlights the results of widespread implementation throughout a region in southwestern Ontario, Canada.” p. 229 | **a)** Nurses  **b)** Non disease specific | **Palliative Performance Scale (PPS) based guidelines** (scored from 0-100. Lower score = greater deterioration):  Mobility level  Capability to perform activities  Disease progression  Capability to attend to personal hygiene  Nutrition and intake of fluids  Level of consciousness  Score of 40% (mainly in bed [incapable of doing majority of activities, extensive disease], mainly assistance needed with self-care [intake is normal or reduced, completely conscious, or drowsy with/without confusion])  Score of 30% (totally bed-bound [incapable of doing any activities, extensive disease, requires full care, intake is normal or reduced, completely conscious, or drowsy with/without confusion]) | | **a)** Starts at admission and frequency of reassessment determined by PPS score however if resident is stable then every 3 months at minimum  PPS score 40%: reassessed every 3 months  PPS score 30%: reassessed every week  **b)** Pathway based on PPS score:  PPS score 40%:  **1.** Educate resident/relatives on likely disease progression  **2.** Support resident/relative/healthcare proxy with needs assessment  **3.** Preparation for dying  PPS score 30%:  **1.** Contemplate the requirement for an MDT meeting including the resident/relatives/health care proxy to discuss goals and expectations  **2.** Converse with resident/relatives about plan of care/ EOL care  **3.** Predict and get ready for likely deterioration of health in the last hours of life  **4.** Medication/treatment review  **5.** Discontinue unnecessary interventions  **6.** Think about other routes of medication administration  **As well as:** Symptom assessment and management (ESAS used) |
| **a)** Henry and Sherwen  (2011)  **b)** UK  **c)** Dying in the “near future” (predicted) | **a)** The National End of Life Care Programme guide  **b)** Narrative article | “The National End of Life Care Programme (NEoLCP) recently published a guide to help staff improve the care of people dying in this setting. This article outlines the guide’s practical approach to this.” p. 137 | **a)** Care home staff and managers  **b)** Non disease specific | | Surprise Question (near future) | **a)** “ongoing basis”  **b)** **1.** Conversing as end of life nears  **2.** Conducting a complete needs assessment, review care goals and preferences, and updating care plan  **3.** Liaising with other services  **4.** Implementing the care plan  **5.** Care in the last days of life  **6.** Care after death  **As well as:** Advance Care Planning, needs assessment, designating a key worker |

**Abbreviations:**

ACD: Advance Care Directive, ACP: Advance Care Planning, ADL: Activities of Daily Living, DNR: Do Not Resuscitate, EOL: End of Life, ESAS: Edmonton Symptom Assessment Scale, GP: General Practitioner, LTC: Long Term Care, MDT: Multidisciplinary Team, NH: Nursing Home, PC: Palliative Care, PPS: Palliative Performance Scale, RACF: Residential Aged Care Facility
